# Antioxidant and anti-inflammatory function of walnut green husk aqueous extract (WNGH-AE) on human hepatocellular carcinoma cells (HepG2) treated with t-BHP

**DOI:** 10.1371/journal.pone.0318005

**Published:** 2025-01-27

**Authors:** Zheng-Qiang Li, Yu-Ting Gao, Chen-Fa Zhao, Rui An, Yan-Lv Wu, Zhi-Pang Huang, Ping Ma, Xu Yang, Rong She, Xiao-Yan Yang

**Affiliations:** 1 Institute of Natural Antioxidants and Anti-Inflammation, Dali University, Dali, Yunnan, China; 2 Institution of Eastern-Himalaya Biodiversity Research, Dali University, Dali, Yunnan, China; 3 Yunling black-and-white snub-nosed monkey observation and research station of Yunnan province, Dali, Yunnan, China; 4 Key Laboratory of Environmental Related Diseases and One Health, Xianning Medical College, Hubei University of Science and Technology, Xianning, Hubei, China; Dr Rammanohar Lohia Avadh University, INDIA

## Abstract

Oxidative damage, oxidative inflammation, and a range of downstream diseases represent significant threats to human health. The application of natural antioxidants and anti-inflammatory agents can help prevent and mitigate these associated diseases. In this study, we aimed to investigate the effectiveness of walnut green husk (WNGH) as an antioxidant and anti-inflammatory agent in an in vitro setting. HepG2 cells were treated with tert-butyl hydroperoxide (t-BHP) to establish a cellular model of oxidative damage and inflammation. We assessed the biocompatibility of walnut green husk aqueous extract (WNGH-AE) on HepG2 cells using MTT and LDH assays (WNGH-AE concentration: 0.05, 0.1, 0.2, 0.4, 0.8, 1.6, 3.2, 6.4, and 12.8 mg/mL). Additionally, we measured intracellular oxidative stress indicators, such as ROS and 8-OHdG, along with inflammatory factors TNF-α and IL-1β through ELISA to evaluate the antioxidant and anti-inflammatory capacity of WNGH-AE (concentration: 0.025, 0.05, 0.1 mg/mL) in HepG2 cells. We also determined the free radical scavenging capacity of various extracts of WNGH using DPPH and ·OH methods. The total phenols, total polysaccharides, and total flavonoids in WNGH-AE were analyzed using the Folin-Ciocalteu’s reagent, the phenol-sulfuric acid method, and the spectrophotometry, respectively. The bioactive components of WNGH-AE were analyzed using LC-MS/MS. Our results demonstrated that WNGH-AE was highly biocompatible with HepG2 cells. The antioxidant effect of WNGH-AE involved the scavenging of intracellular ROS, while its anti-inflammatory effect was linked to the down-regulation of the NF-κB pathway. Compared to other extractants (ethyl acetate, n-butanol, 75% ethanol, and petroleum ether), WNGH-AE exhibited the strongest free radical scavenging ability. Through LC-MS/MS analysis, we identified 403 compounds in WNGH-AE, with gentisic acid being the most abundant and possessing high antioxidant capacity, suggesting it may be a key active component contributing to the antioxidant activity of WNGH-AE. In conclusion, our findings indicate that WNGH-AE is a natural, high-quality antioxidant and anti-inflammatory biomaterial deserving further research and development, with significant potential applications in healthcare. (311 words)

## 1. Introduction

Oxidative damage and oxidative inflammation pose significant threats to human health [1]. Oxidative stress refers to the generation of intracellular reactive oxygen species (ROS) that exceed the capacity of cells to eliminate them, resulting in a redox imbalance [2]. The accumulation of ROS leads to cellular and biomolecular damage, a phenomenon known as oxidative damage [3]. Furthermore, the excessive accumulation of ROS activates the NF-κB pathway [4], which triggers the synthesis and release of inflammatory factors, resulting in inflammation in cells and tissues—termed oxidative inflammation [5]. The onset of oxidative damage and oxidative inflammation is often linked to a cascade of downstream diseases, with cardiovascular diseases, cancers, diabetes, and other chronic non-communicable diseases (CNCDs) being among the most prevalent, thereby posing serious risks to human health [6, 7]. In contrast to synthetic alternatives, natural antioxidant products are generally safer, devoid of side effects, and exhibit more potent antioxidant effects [8]. Consequently, researchers worldwide are actively investigating efficient and natural antioxidant and anti-inflammatory substances. Notable natural antioxidant products have been identified from both plant and animal sources, including spirulina [9] and natural astaxanthin [10]. However, the high costs associated with their limited production underscore the necessity for continued exploration of high-quality natural antioxidant substances, particularly those derived from readily available and inexpensive raw materials.

Walnut green husks possess a variety of biological activities and are an underestimated byproduct of walnut nut production [11]. Known as green dragon clothes, walnut green husks have demonstrated antitoxic, anti-swelling, analgesic, and anti-inflammatory effects in traditional Chinese medicine [12–15]. These effects are primarily attributed to the presence of a diverse array of bioactive substances recognized for their antioxidant properties, including polysaccharides [16], phenols [17], and ketones [13]. Consequently, walnut green husks hold significant potential for development into high-quality natural antioxidants and anti-inflammatory products. Research has shown that extracts from walnut green husks exhibit robust free radical scavenging abilities [18] and can reduce inflammatory factor levels in the livers of mice with non-alcoholic steatohepatitis [19], among other bioactive benefits [20–23]. However, there is a notable absence of anti-inflammatory capacity assessments at the cellular level, which somewhat limits the broader application of these extracts in practical contexts. Furthermore, within the global walnut industry, walnut nuts are the primary focus of utilization [24], leading to the neglect and waste of substantial quantities of walnut green hull by-products [25]. Therefore, advancing the development of walnut green husks into high-quality natural antioxidants is anticipated to address the current limitations in the production of natural antioxidant substances, particularly yield constraints.

The t-BHP induced oxidative stress cell model in HepG2 cells: HepG2 cells, derived from human hepatocellular carcinoma, are extensively utilized in the research and development of natural antioxidants and anti-inflammatory agents [26]. Tert-butyl hydroperoxide (t-BHP) is a widely employed organic peroxide that facilitates various reactions, including oxidation, epoxidation, and decarboxylation. In the context of investigating natural antioxidants and anti-inflammatory compounds, t-BHP serves as a tool to induce oxidative stress in HepG2 cells, thereby establishing oxidative stress cell models. Currently, the application of these oxidative stress cell models is fundamental in evaluating the antioxidant capacity of potential substances.

**Fig 1:**
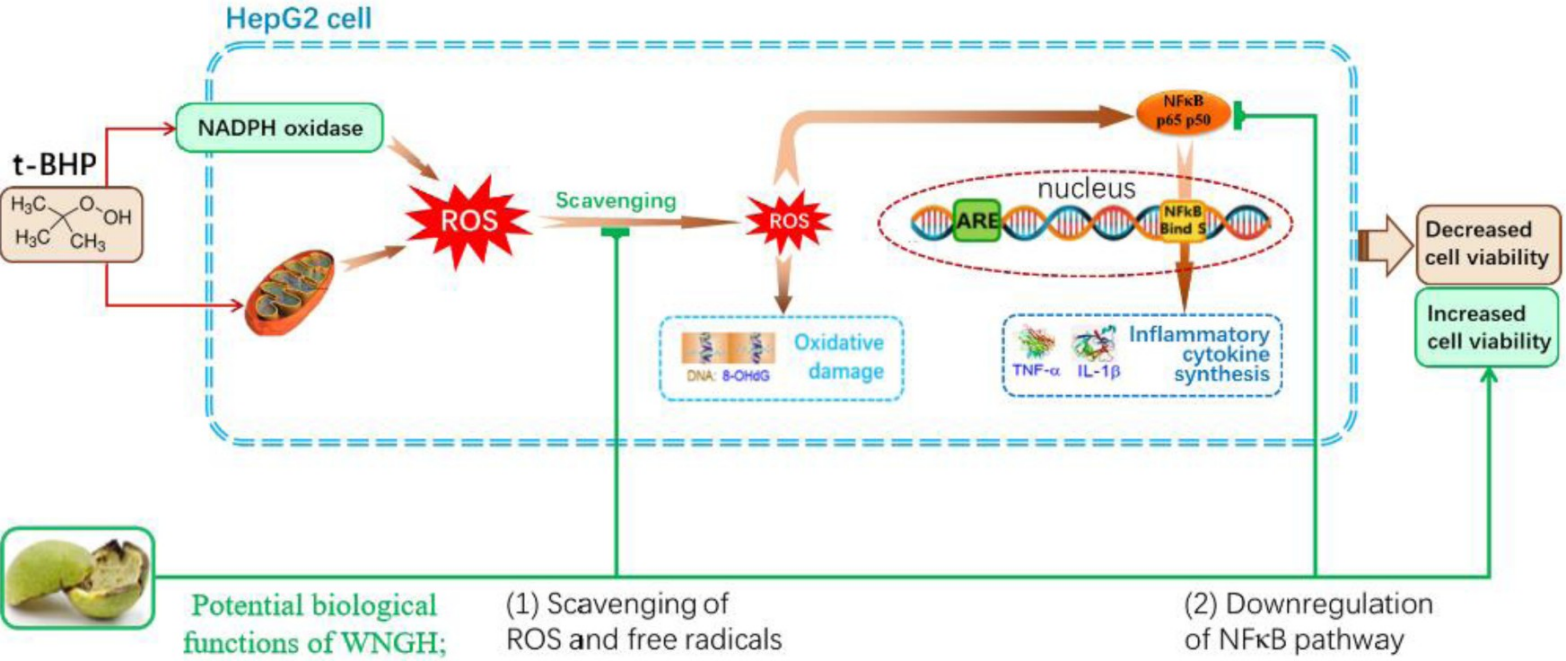
The scientific hypothesis of this study.

The scientific hypothesis of this study: WNGH-AE possesses potential biological functions, which can (1) prevent oxidative stress by scavenging ROS and free radicals; (2) prevent oxidative inflammation by downregulating the NF-κB pathway; (3) increase cell viability, thereby protecting cells. To enhance the scientific rigor of our research, we have formulated the hypothesis in Fig 1.

## 2. Materials and methods

### 2.1 Agents and instruments

All chemical reagents used in this study were of analytical grade. Folin reagent, phenol, petroleum ether, ethyl acetate, anhydrous sodium acetate, n-butanol, 95% ethanol, sodium carbonate, sodium nitrite, aluminum nitrate (Al(NO_3_)_3_), sodium hydroxide, and sulfuric acid were obtained from National Pharmaceutical Chemical Reagent Co., Ltd. Acetonitrile was sourced from DIKMA, while formic acid was acquired from TCI. Additionally, 1,1-diphenyl-2-trinitrophenylhydrazine (DPPH) and tert-butyl hydroperoxide (t-BHP) were purchased from Shanghai McLean Biochemical Technology Co., Ltd. The DMEM high glucose culture medium was procured from Gibco, USA. The ammonium format along with ascorbic acid (VC) were obtained from Sigma-Aldrich. The 3-(4,5-dimethylthiazole-2)-2,5-diphenyltetrazolium bromide (MTT) test kit, 8-OHdG test kit, ROS reagent kit, and NF-κB assay kit were purchased from Shanghai Enzyme Linked Biotechnology Co., Ltd. Finally, the IL-1β assay kit and TNF-α assay kit were sourced from Boster Bioengineering Co., Ltd. in Wuhan, China.

The study utilized several instruments, including a double distilled deionized water system (laboratory purified water system, Shanghai Hitech Instrument Co., Ltd.), an electronic balance (PX224ENH, Germany OHAUS Instrument Co., Ltd.), a carbon dioxide incubator (HF90, Shanghai Heli Biopharmaceutical Holdings Co., Ltd.), an inverted biological microscope (BDS500, Chongqing Optec Instrument Co., Ltd.), an ELISA microplate analyzer (DNM-9602G, Beijing Perlove Co., Ltd.), a rotary evaporator (RE-2000A, Shanghai Hitech Instrument Co., Ltd.), an ultrasonic extractor (SB25-12DDT, Ningbo Xinzhi Biotechnology Co., Ltd.), and a freeze-drying instrument (SCIENTZ-10N, Ningbo Xinzhi Biotechnology Co., Ltd.), an ultra-high performance liquid chromatography (UPLC) (ACQUITY, Waters, Milford), an ACQUITY UPLC® HSS T3 column (2.1×150 mm, 1.8 μm) (Waters, Milford), an Thermo Q Exactive (Thermo Fisher Scientific).

### 2.2 Experimental protocol

**Fig 2:**
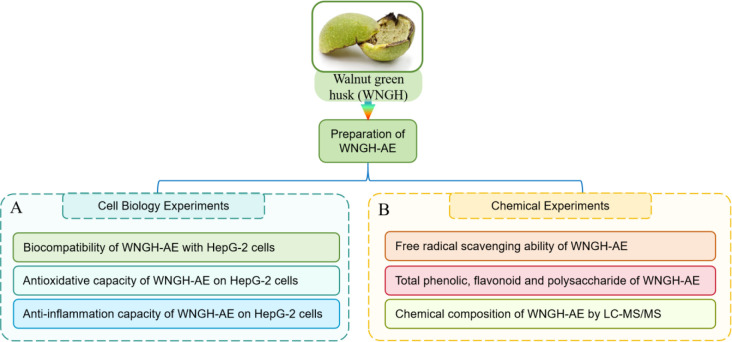
The experimental protocol of this study. (A) Cell biological experiments; (B) Chemical experiments.

The experimental protocol of this study is shown in Fig 2.

### 2.3 Preparation of experimental samples

**Fig 3:**
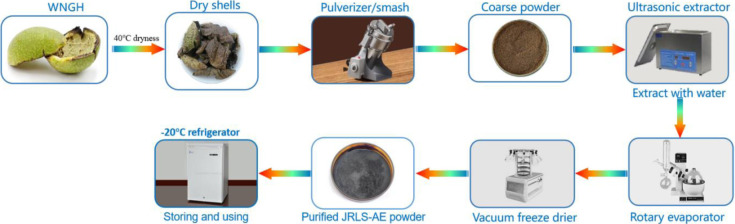
The extraction process of WNGH-AE.

Walnut green husks were collected in May 2022 from the Dali University Ancient City Campus in Dali Town, Dali City, Yunnan Province, China (E: 100° 9′ 0.90″; N: 25° 40′ 15.96″, altitude 2200 m). The collected walnut was identified by Dr. Li Ji-Hong of the Institute of Eastern-Himalaya Biodiversity Research, Dali University. Following fruit collection, the walnut green skin was immediately separated and dried at 40°C for 48 hours. It was then crushed and sieved through a 100-mesh sieve, and the resulting powder was set aside for later use. A total of 50 grams of walnut green skin powder was placed in a 500-milliliter conical flask, to which 500 milliliters of petroleum ether was added, maintaining a 1:10 ratio of material to liquid. The mixture was adjusted to a temperature of 20°C and subjected to ultrasonic extraction at 40 Hz for 1 hour. After extraction, it was allowed to stand in the dark for 24 hours. The supernatant was then collected, filtered under vacuum, and the filtrate was concentrated using a rotary evaporator. This process was repeated using ethyl acetate, n-butanol, 75% ethanol, and distilled water in place of petroleum ether, yielding ethyl acetate extract, n-butanol extract, 75% ethanol extract, and walnut husk water extract, respectively. The extracts were stored at -20°C for later use (Fig 3).

### 2.4 Cell biocompatibility of WNGH-AE

#### 2.4.1 Cell line and its cultivation

HepG2 cells were obtained from the Kunming Cell Culture Bank at the Chinese Academy of Sciences (cell number: KCB200507YJ). Prior to the formal experiments, the cell line was verified for authenticity using the International Cell Line Authentication Committee (ICLAC) guidelines [27]. During cell culture, the cells were seeded into a 75 cm^2^ culture flask containing 10 mL of culture medium and incubated in a high-humidity environment at 37°C with 5% CO_2_. The culture medium was replaced every 24 hours. This medium consisted of 84% DMEM high glucose medium, 15% fetal bovine serum, and a mixture of 1% penicillin (1000 U/mL) and streptomycin (1000 μg/mL). Subculturing was performed once the cells reached 80% to 90% confluence on the flask bottom [28].

#### 2.4.2 Determination of biocompatibility of WNGH-AE with HepG2 cells

The MTT assay and LDH release assay were employed to assess the biocompatibility of WNGH-AE with HepG2 cells. HepG2 cells were seeded into a 96-well cell culture plate at a density of 1×10^5^ cells per well. Following 2 days of culture, the cells were treated with various concentrations of WNGH-AE (0.05, 0.1, 0.2, 0.4, 0.8, 1.6, 3.2, 6.4, and 12.8 mg/mL) for a duration of 24 hours. The MTT assay was utilized to evaluate cell viability (batch number: 16H12B56, Shanghai Enzyme Chain Biotechnology Co., Ltd., China) [29], while the LDH assay was conducted to quantify the lactate dehydrogenase (LDH) released by the cells (batch number: A020-2-2, Nanjing Jiancheng Bioengineering Institute). Absorbance was measured at 450 nm using an enzyme-linked immunosorbent assay (ELISA) reader.

### 2.5 Determination of antioxidant and anti-inflammatory function of WNGH-AE

The results of the WNGH-AE cytotoxicity testing indicated that concentrations exceeding 0.8 mg/mL exerted toxic effects on HepG2 cells. To more effectively demonstrate the biological activity of WNGH-AE while minimizing experimental costs, the drug concentrations were adjusted to 0.025, 0.05, and 0.1 mg/mL. The effect of WNGH-AE on the antioxidant stress resistance of HepG2 cells was evaluated using reactive oxygen species (ROS) and 8-hydroxydeoxyguanosine (8-OHdG) as indicators. Additionally, the impact of WNGH-AE on the antioxidant and inflammatory capacities of HepG2 cells was assessed using nuclear factor kappa B (NF-κB), tumor necrosis factor alpha (TNF-α), and interleukin-1 beta (IL-1β) as biomarkers. Using Vitamin C serves as a positive control for the assessment of the antioxidant capacity of WNGH-AE, thereby measuring the level of antioxidant and anti-inflammatory effects of WNGH-AE. The concentration of HepG2 cells was adjusted to 5×10^5^ cells/mL, and 2.0 mL of the cell suspension was inoculated into each well of a 6-well plate, followed by incubation in a high-humidity incubator at 37°C with 5% CO_2_. The culture medium was replaced every 12 hours. After 2 days, the cell culture medium was discarded, and each well was washed twice with 1 mL of phosphate-buffered saline (PBS). Subsequently, HepG2 cells were pre-treated with varying concentrations (0.0, 0.025, 0.05, 0.1 mg/mL) of WNGH-AE and 1 mg/mL of vitamin C (VC) for 12 hours, with six replicates per group. The optimal method for evaluating the antioxidant effects of WNGH-AE is to employ a HepG2 cell model with features of oxidative stress and inflammation. Hence, we proactively investigated the conditions required for constructing this pathological model., specifically: The cells were then treated with 0.2 μmol/L tert-butyl hydroperoxide (t-BHP) for an additional 12 hours. WNGH-AE, t-BHP, and VC were all diluted using Dulbecco’s Modified Eagle Medium (DMEM). After 5 hours, 200 μL of culture medium was extracted from each well into a 1.5 mL centrifuge tube, sealed, and stored at -20°C for the subsequent determination of ROS, 8-OHdG, NF-κB, TNF-α, and IL-1β. The quantification of ROS, 8-OHdG, and NF-κB was performed using respective assay kits (batch numbers: No.202402, No.02/2024, and YJ345112, Shanghai Enzyme Linked Biotechnology Co., Ltd., China). The quantification of TNF-α and IL-1β was conducted using TNF-α and IL-1β assay kits (batch numbers: No. 23918114215 and No. 11418110215, Wuhan Boster Bioengineering Co., Ltd.).

### 2.6 Free radical scavenging ability of WNGH-AE

#### 2.6.1 DPPH assay

The DPPH radical scavenging assay was performed according to the methodology described in the literature [30]. First, to construct a standard curve for calculating the free radical scavenging rate of the sample, the absorbance of DPPH was measured at concentrations of 5, 10, 20, 40, 60, 80, and 100 mg/mL. Second, all extracts were diluted with anhydrous ethanol, except for the WNGH aqueous extract (WNGH-AE), which was diluted with water, to prepare solutions with varying concentrations (0.025, 0.05, 0.1, 0.2, 0.4, 0.8, 1.6, and 3.2 mg/mL). Third, a 50 mg/mL DPPH solution prepared in anhydrous ethanol was mixed with the WNGH extracts at different concentrations in a 1:1 ratio (sample). Anhydrous ethanol was used as the control (sample control), and ethanol mixed with DPPH served as the blank (blank control). After incubating the mixtures at 37°C in the dark for 30 minutes, the absorbance was measured at 517 nm. Each sample was tested in five replicates, alongside one sample control and one blank control. Finally, the DPPH free radical scavenging activity of the WNGH extracts was calculated using the following formula:

$$DPPH\;radical\;scavenging\;activity=(1-\frac{A_{Sample}-A_{Control}}{A_{Blank}})\times 100\%$$

In the above formula: A_*Sample*_ is the absorbance of the sample tube; A_*Control*_ is the absorbance of the control tube; A_*Blank*_ is the absorbance of the blank tube.

#### 2.6.2 Hydroxyl radical (·OH) assay

The hydroxyl (·OH) scavenging experiment was conducted according to the methodology outlined by Lin et al. [31]. With the exception of WNGH-AE, which was diluted with water, all other extracts were diluted with anhydrous ethanol and prepared into solutions of varying concentrations (0.1, 0.2, 0.4, 0.8, 1.6, 3.2, 6.4, 12.8 mg/mL). For the WNGH sample, it was mixed with 3 μmol/mL FeSO_4_ and 3 μmol/mL H_2_O_2_ in a ratio of 1:1:1. The reaction was conducted in the dark at 25°C for 10 minutes, after which salicylic acid was added and the mixture was allowed to react in the dark at 25°C for an additional 30 minutes (Sample). Anhydrous ethanol was used as a control in place of salicylic acid, while anhydrous ethanol was also used as the blank in place of the sample. The absorbance of the reaction mixture was measured at 517 nm, with five replicates set for each sample, one sample control, and one blank control. The formula for calculating the ·OH scavenging activity of WNGH is as follows:

$$\cdot OH\;radical\;scavenging\;activity=(1-\frac{A_{Sample}-A_{Control}}{A_{Blank}})\times 100\%$$

In the above equation: A_*Sample*_ is the absorbance of the sample tube; A_*Control*_ is the absorbance of the control tube; A_*Blank*_ is the absorbance of the blank tube.

### 2.7 Determination the content of bioactive compounds

#### 2.7.1 Measurement of total phenolic, total flavonoid and polysaccharide content

The total phenolic content was quantified using Folin–Ciocalteu’s reagent with gallic acid as the standard [32]. Briefly, 1 mL of a 1 g/mL WNGH-AE solution was mixed with 5 mL of 10% Folin–Ciocalteu’s reagent in a test tube. After allowing the reaction to proceed for 8 minutes, 4 mL of 7.5% Na_2_CO_3_ was added. The mixture was then thoroughly mixed and allowed to react for 1 hour in the dark. The absorbance was measured at 765 nm, and the total phenolic content is expressed as gallic acid equivalents (μg GAE/mg).

The total flavonoid content was quantified using rutin as the standard, following the reference method outlined by Zhisheng et al. [33]. In brief, 1 mL of a 1 g/mL WNGH-AE solution was combined with 600 μL of a 1:1 (v/v) mixture of 5% (w/v) NaNO_2_ and 10% Al (NO_3_)_3_. The reaction proceeded as follows: 1 mL of 1 mol/L NaOH and 3.4 mL of 30% ethanol were added and mixed well, followed by a 6-minute incubation. The mixture was then allowed to react in the dark for 15 minutes, after which the absorbance was measured at 510 nm. The total flavonoid content was expressed in rutin equivalents (μg RE/mg).

Using rutin as a standard, the total polysaccharide content was determined via the phenol-sulfuric acid method [34]. Briefly, 1 mL of 1 g/mL WNGH-AE was mixed with 1 mL of 5% phenol and 5 mL of 1.84 g/mL H_2_SO_4_, and the mixture was incubated in the dark for 30 minutes. The absorbance was subsequently measured at 490 nm. The total polysaccharide content was expressed in glucose equivalents (μg GE/mg).

#### 2.7.2 LC-MS analysis

WNGH-AE lyophilized material was sent to Suzhou Panomics Biomedical Technology Co. for LC-MS analysis. Please read the detailed procedure in Annex 1 (S1 File).

### 2.8 Statistical analysis

All data in this study were analyzed using one-way analysis of variance (ANOVA), followed by the least significant difference (LSD) test. The data were processed using GraphPad Prism 7 software (San Diego, CA, USA) and are presented as mean ± standard deviation (SD). A p-value of less than 0.05 was considered statistically significant.

## 3. Results

### 3.1 Biocompatibility of WNGH-AE with HepG2 cells

Based on the following considerations, we will focus solely on testing the antioxidant activity of the walnut green husk water extract (WNGH-AE) in subsequent experiments: (1) Among the WNGH extracts obtained from various solvents, WNGH-AE demonstrates the most effective free radical scavenging activity; (2) To achieve the most accurate evaluation of the antioxidant and anti-inflammatory properties of the extract, it is essential to ensure that the sample is free from solvent toxicity. Furthermore, for practical applications in later stages, it is preferable that the extraction reagents used are both universal and non-toxic; (3) The medicinal properties of walnut green husk primarily emphasize its aqueous extract.

**Fig 4:**
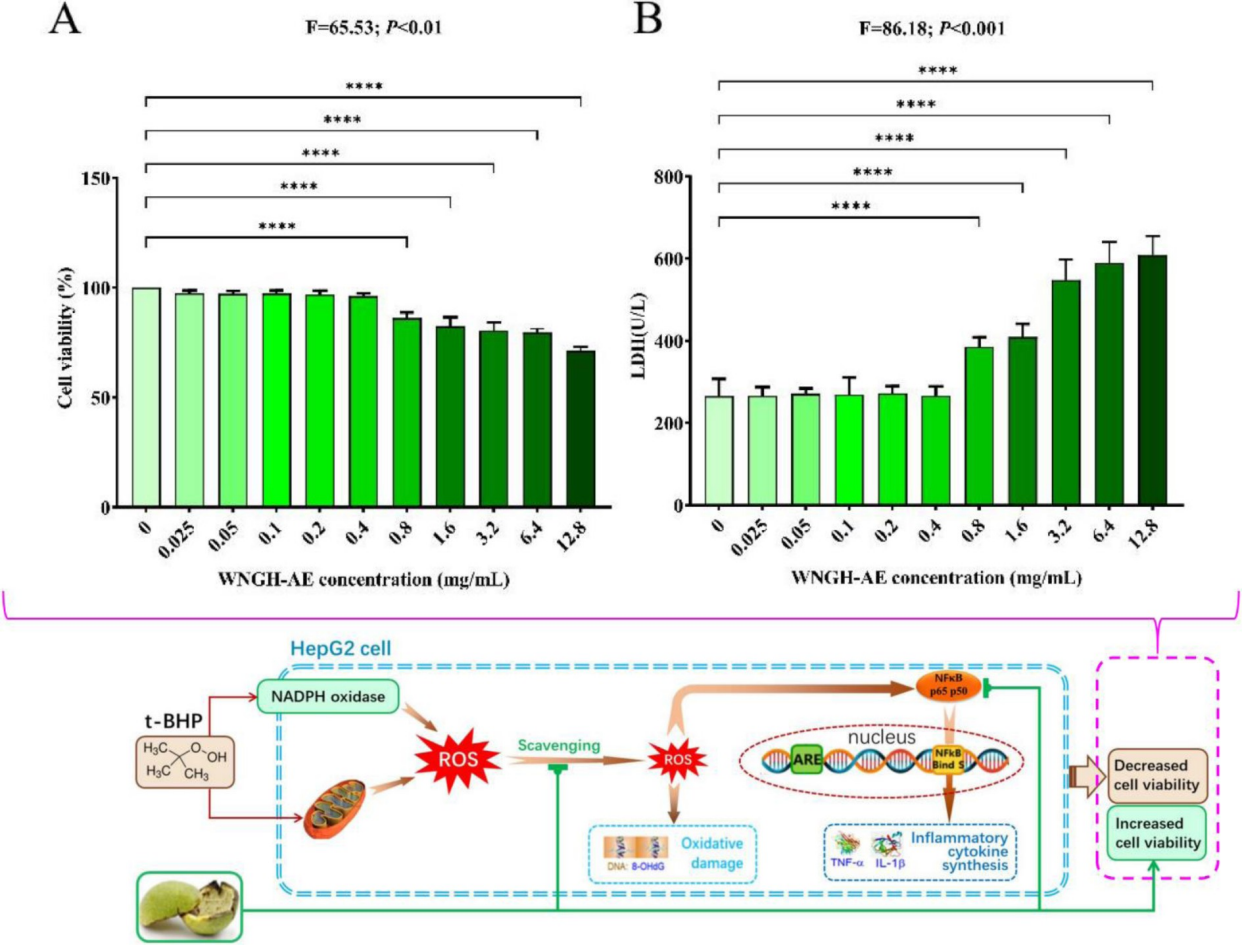
Cytocompatibility of different concentrations of WNGH-AE. (A) MTT value (%) of different concentrations of WNGH-AE; (B) LDH value (U/L) of different concentrations of WNGH-AE. (****: indicates *P*<0.001, comparison with t-BHP group).

To evaluate biocompatibility, we treated HepG2 cells with various concentrations of WNGH-AE for 24 hours and subsequently assessed changes in cell viability. The results of the MTT assay indicated that, compared to the cells in the control group (0.00 mg/mL), there was no statistically significant difference in cell viability when the concentration of WNGH-AE ranged from 0.025 to 0.4 mg/mL. However, a statistically significant difference (*P*<0.001) was observed at concentrations between 0.8 and 12.8 mg/mL. This evidence suggests that low concentrations (0.0 to 0.4 mg/mL) of WNGH-AE do not affect HepG2 cells, whereas higher concentrations (0.8 to 12.8 mg/mL) exhibit cytotoxic effects on HepG2 cells (Fig 4A, F = 65.53, *P*<0.01).

HepG2 cells were treated with varying concentrations of WNGH-AE for 24 hours, and changes in lactate dehydrogenase (LDH) release were assessed. The results indicated that there was no significant change in LDH release at WNGH-AE concentrations ranging from 0.025 to 0.4 mg/mL. However, within the concentration range of 0.8 to 12.8 mg/mL, a significant increase in LDH release was observed compared to the control group (0.0 mg/mL) (*P*<0.001). This finding suggests that high concentrations of WNGH-AE (0.8 to 12.8 mg/mL) induce toxic effects on the cells, resulting in cell damage and increased LDH release (Fig 4B, F = 86.18, *P*<0.001).

### 3.2 Anti-oxidative stress capacity of WNGH-AE on HepG2 cells

When the body is in a state of oxidative stress, it produces excessive reactive oxygen species (ROS). Prolonged oxidative stress can trigger an inflammatory response. Free radicals activate signaling pathways such as NF-κB and SP1, leading to the production of inflammatory factors, including IL-1β and TNF-α, which can ultimately result in various chronic diseases. Furthermore, when ROS attack biomolecules such as lipids and DNA, they generate damage products like malondialdehyde (MDA) and 8-hydroxydeoxyguanosine (8-OHdG), which often promote abnormal cell proliferation and significantly increase the likelihood of cancer development. Consequently, this study selected ROS and 8-OHdG as biomarkers of oxidative stress, with NF-κB indicating the activation level of inflammatory pathways, and IL-1β and TNF-α serving as inflammatory biomarkers. This approach aims to evaluate the effects of WNGH-AE on the antioxidant stress and anti-inflammatory capacity of HepG2 cells.

**Fig 5:**
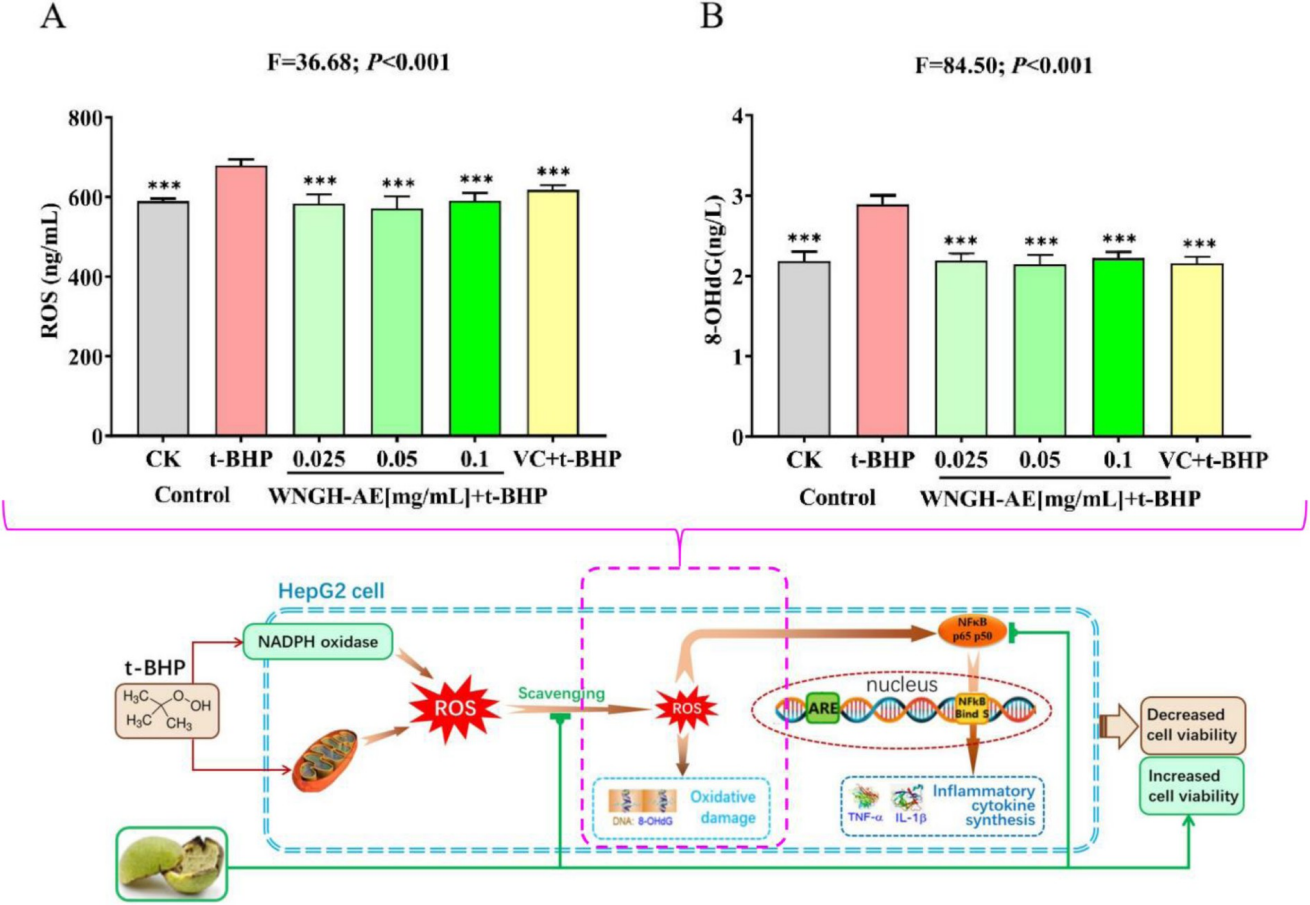
Levels of the effect of WNGH-AE on ROS levels in HepG2 cells. (A) Detection of cellular ROS level; (B) Detection of cellular 8-OHdG level; (* Indicates comparison with t-BHP group, **: *P*<0.01, ***: *P*<0.001.).

The experimental results demonstrated that treatment with 0.2 μmol/mL t-BHP for 12 hours significantly increased the ROS levels in HepG2 cells by 15.29% (*P*<0.001) compared to the blank control group. This indicates that t-BHP treatment can induce an oxidative stress response in HepG2 cells. In comparison to the t-BHP group, cells that were pre-treated with VC for 12 hours and then exposed to 0.2 μmol/mL t-BHP exhibited a 9.17% reduction in ROS levels (*P*<0.001), suggesting that VC has the capacity to scavenge ROS. Furthermore, when cells were pre-treated with WNGH-AE at varying concentrations (0.025, 0.05, and 0.1 mg/mL) for 12 hours prior to treatment with 0.2 μmol/mL t-BHP, the ROS levels decreased by 14.07%, 15.94%, and 13.10%, respectively, compared to the t-BHP group (*P*<0.001 for all). These findings indicate that WNGH-AE, at concentrations ranging from 0.025 to 0.1 mg/mL, serves as a highly effective and promising antioxidant stress agent (Fig 5A).

After treatment with 0.2 μmol/mL t-BHP for 12 hours, the level of 8-OHdG in HepG2 cells increased by 32.24% (*P*<0.001) compared to the blank control group, indicating that t-BHP treatment induces DNA damage in HepG2 cells. In contrast, cells pre-treated with VC for 12 hours prior to exposure to 0.2 μmol/mL t-BHP for the same duration exhibited a 1.41% reduction in 8-OHdG content (*P*>0.05), suggesting that VC has minimal protective effects against t-BHP-induced DNA damage in HepG2 cells. Furthermore, after 12 hours of pre-treatment with WNGH-AE at varying concentrations (0.025, 0.05, and 0.1 mg/mL), the 8-OHdG levels in cells treated with these concentrations decreased by 24.11%, 25.83%, and 23.22%, respectively, compared to the t-BHP group (*P*<0.001 for all). Notably, the efficacy of the 0.025, 0.05, and 0.1 mg/mL treatment groups was significantly greater than that of VC in preventing t-BHP-induced DNA damage in HepG2 cells (*P*<0.001), indicating that WNGH-AE is a highly promising agent for mitigating DNA damage and suggesting its potential as an antioxidant agent (Fig 5B).

### 3.3 Effect of WNGH-AE on the anti-inflammatory capacity

**Fig 6:**
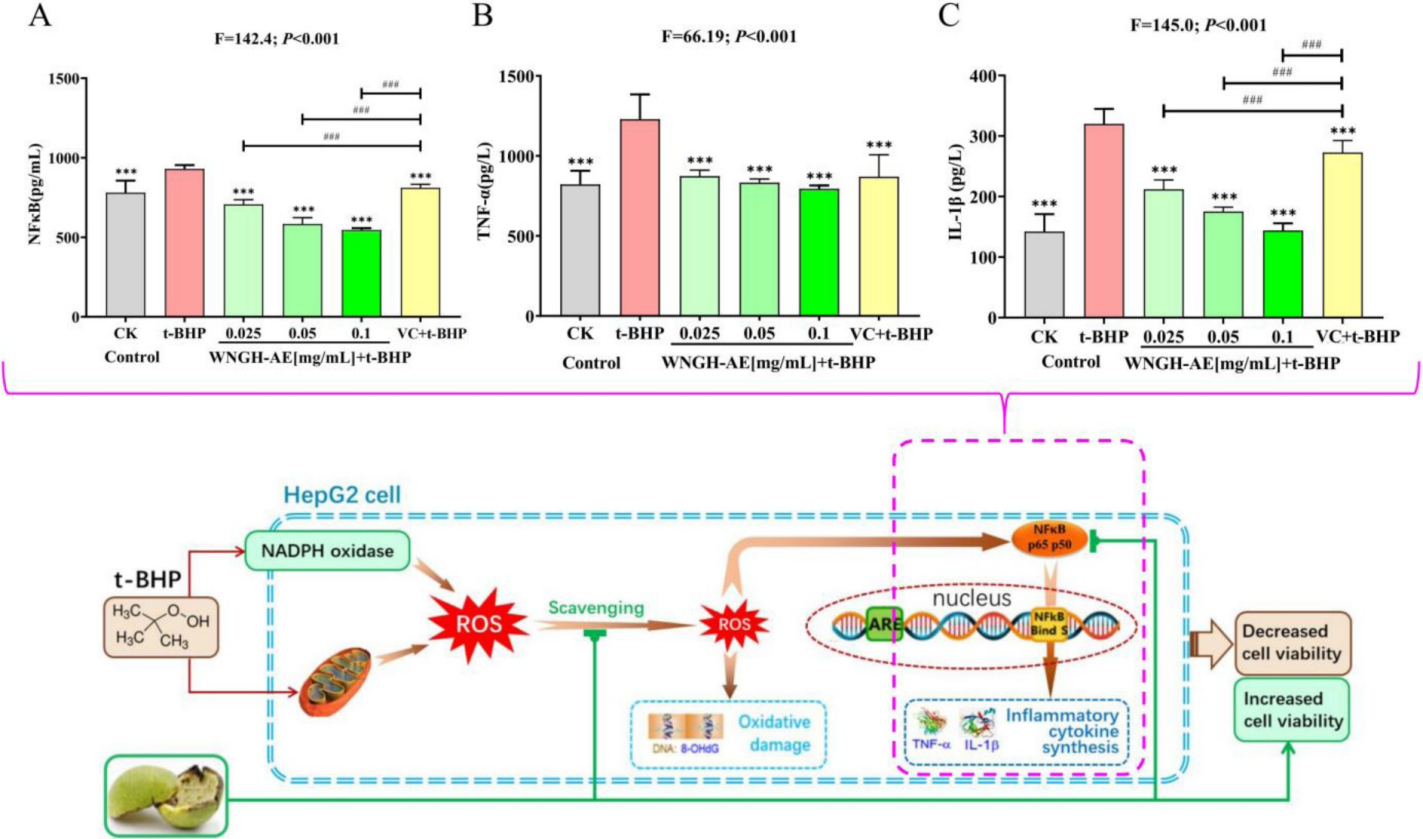
Effect of WNGH-AE on the anti-oxidative stress capacity of HepG2 cells. (A) Detection of cellular NF-κB level; (B) Detection of cellular TNF-α level; (C) Detection of cellular IL-1β level. (* Indicates comparison with t-BHP group, **: *P*<0.01, ***: *P*<0.001; # numbers indicate comparison with VC group, ###: *P*<0.001.).

After treatment with 0.2 μmol/mL t-BHP for 12 hours, the NF-κB content in HepG2 cells significantly increased by 5.09% (*P*<0.001) compared to the blank control group. This finding indicates that t-BHP treatment can upregulate NF-κB in HepG2 cells and trigger inflammatory responses. When cells were pre-protected with VC for 12 hours and subsequently treated with 0.2 μmol/mL t-BHP, their NF-κB content decreased by 12.77% (*P*>0.05) compared to the t-BHP group, suggesting that VC had no anti-inflammatory effect. Conversely, when cells were pre-protected with WNGH-AE at different concentrations (0.025, 0.05, and 0.1 mg/mL) for 12 hours before treatment with 0.2 μmol/mL t-BHP, the NF-κB content in the 0.025, 0.05, and 0.1 mg/mL treatment groups decreased significantly by 23.99%, 37.24%, and 41.27%, respectively, compared to the t-BHP group (*P*<0.001 for all). Furthermore, 0.025, 0.05, and 0.1 mg/mL WNGH-AE demonstrated significantly greater downregulation of NF-κB signaling than VC (*P*<0.001 for all). WNGH-AE effectively reduces the inflammatory response induced by t-BHP in cells by downregulating the NF-κB pathway, exhibiting excellent anti-inflammatory effects (Fig 6A)

After treatment with 0.2 μmol/mL t-BHP for 12 hours, the level of TNF-α in HepG2 cells increased by 49.53% (*P*<0.001) compared to the blank control group, indicating that t-BHP treatment can induce an oxidative inflammatory response in HepG2 cells. In comparison to the t-BHP group, cells that were pre-protected with VC for 12 hours and subsequently treated with 0.2 μmol/mL t-BHP exhibited a 29.23% decrease in TNF-α levels (*P*<0.001), suggesting that VC possesses significant antioxidant and anti-inflammatory properties. Furthermore, cells pre-protected with WNGH-AE at various concentrations (0.025, 0.05, and 0.1 mg/mL) for 12 hours before treatment with 0.2 μmol/mL t-BHP demonstrated decreases in TNF-α levels of 28.92%, 32.23%, and 35.37%, respectively (*P*<0.001 for all concentrations). These findings indicate that WNGH-AE, at concentrations of 0.025, 0.05, and 0.1 mg/mL, is a highly promising anti-oxidative inflammatory agent (Fig 6B).

After treatment with 0.2 μmol/mL t-BHP for 12 hours, the level of IL-1β in HepG2 cells increased by 125.27% (*P*<0.001) compared to the blank control group, indicating that t-BHP treatment can induce an oxidative inflammatory response in HepG2 cells. In comparison to the t-BHP group, cells pre-treated with VC for 12 hours and subsequently exposed to 0.2 μmol/mL t-BHP exhibited a significant decrease of 14.80% (*P*<0.001) in IL-1β levels, suggesting that VC possesses the ability to reduce IL-1β. Furthermore, when cells were pre-treated with WNGH-AE at varying concentrations (0.025, 0.05, and 0.1 mg/mL) for 12 hours prior to treatment with 0.2 μmol/mL t-BHP, IL-1β levels decreased by 33.79%, 45.22%, and 55.06%, respectively (*P*<0.001 for all comparisons). Additionally, WNGH-AE at concentrations of 0.025, 0.05, and 0.1 mg/mL demonstrated significantly superior efficacy in reducing IL-1β levels compared to VC (*P*<0.001 for all comparisons). Thus, WNGH-AE at these concentrations can effectively mitigate cell inflammation induced by t-BHP, positioning it as a highly anti-oxidative inflammatory agent (Fig 6C).

### 3.4 Free radical scavenging activity of WNGH

**Fig 7:**
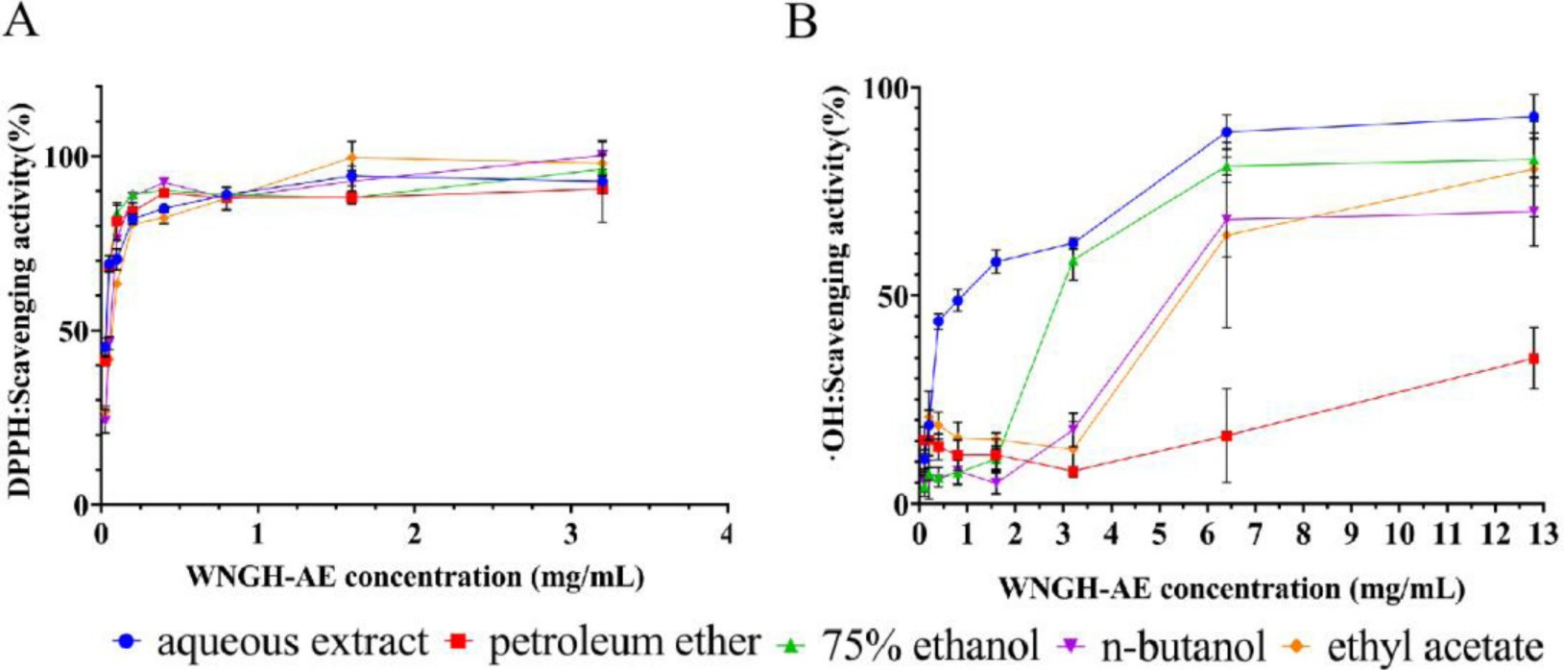
Antioxidant activity of WNGH extracts. (A). Scavenging activity of different solvent extracts of WNGH against DPPH; (B). Scavenging activity of different solvent extracts of WNGH against ·OH.

The antioxidant activity of various solvent extracts was evaluated through DPPH radical and ·OH radical scavenging experiments. The results indicated that the WNGH extracts, prepared using five solvents (petroleum ether, ethyl acetate, n-butanol, 75% ethanol, and distilled water), displayed a concentration-dependent trend in its ability to scavenge both DPPH and ·OH radicals (Fig 7). Based on the IC50 values, the relative strength of the five extracts for DPPH radical scavenging activity was as follows: aqueous extract > petroleum ether extract = 75% ethanol extract > n-butanol extract > ethyl acetate extract. The order of strength for ·OH radical scavenging activity was: aqueous extract > 75% ethanol extract > ethyl acetate extract > n-butanol extract > petroleum ether extract (Table 1). Overall, the antioxidant activity of the WNGH water extract (WNGH-AE) was found to be the most effective.

**Table 1:** Semi-inhibitory activity (IC50) of solvent extracts of WNGH against DPPH and ·OH radicals.

Samples for Extracting	IC50 (mg/mL)	
	DPPH assay	·OH assay
WNGH—distilled water extract	0.027	0.96
WNGH—75% ethanol extract	0.028	3.83
WNGH—ethyl acetate extract	0.064	5.74
WNGH—n-butanol extract	0.048	7.42
WNGH—petroleum ether extract	0.028	24.56

### 3.5 Determination of flavonoid, polyphenol and total sugar contents

We analyzed the total phenols, total polysaccharides, and total flavonoids in WNGH-AE. The results indicated that the polysaccharide content was relatively high, measuring 130.58±0.40 μg GE/mg. In contrast, the contents of phenolics and flavonoids were notably low, recorded at 0.1069±0.11 μg GAE/mg and 0.0140±0.43 μg RE/mg, respectively (Table 2).

**Table 2:** Total phenolic, flavonoid and polysaccharide content of WNGH-AE.

Chemical Composition	Measurement (mean±SD)
Total phenolic content (TPC) (μg GAE/mg)	0.1069±0.11
Total flavonoid content (TFC) (μg RE/mg)	0.0140±0.43
Total polysaccharide content (μg GE/mg)	130.58±0.40

**Fig 8:**
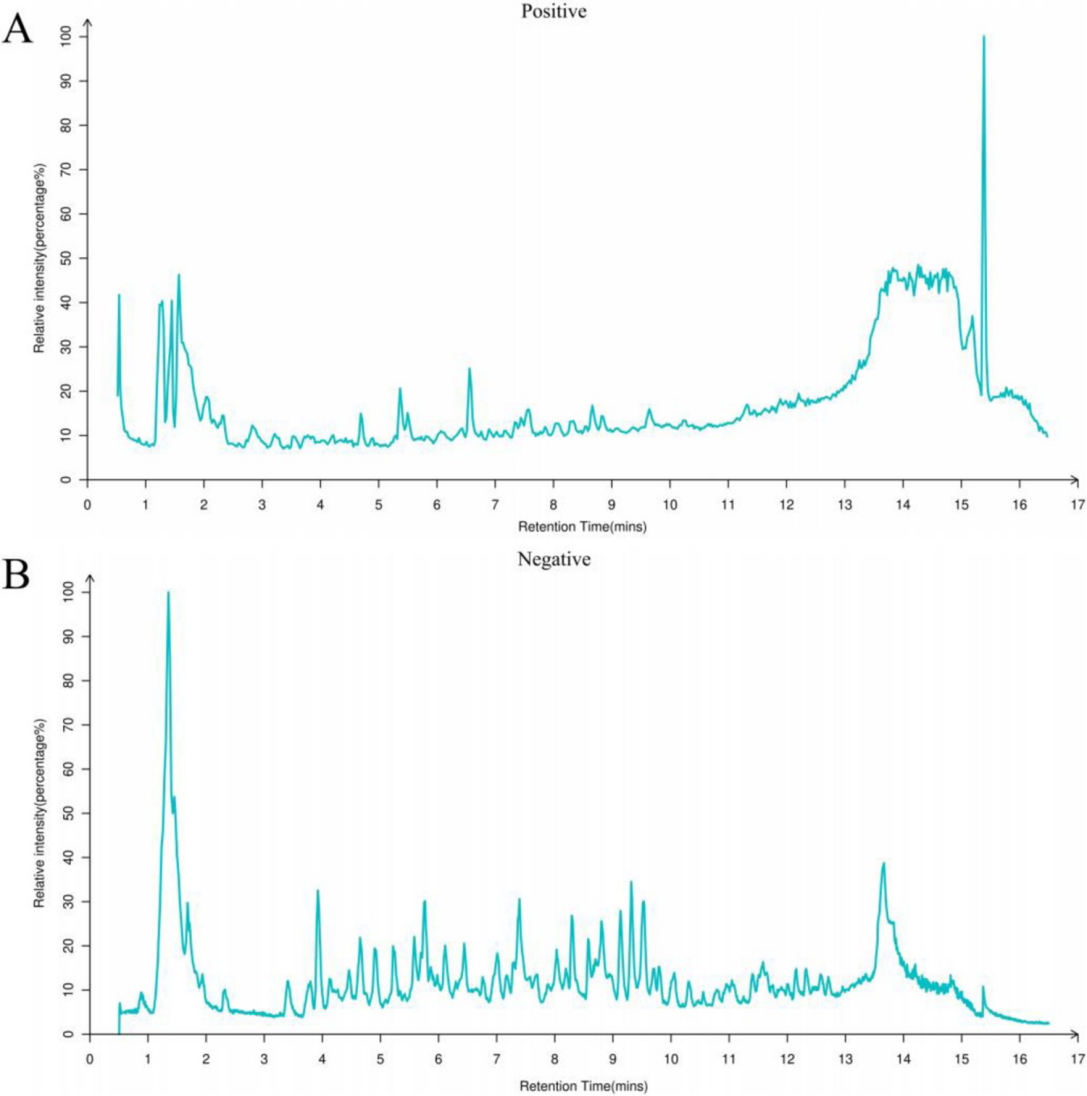
LC -MS analysis of WNGH-AE. (A): Positive ion flow diagram; (B): Negative ion flow diagram.

Non-targeted UPLC-MS/MS analysis was employed to determine the chemical composition of WNGH-AE. A total of 403 compounds were identified, with 281 detected in positive ion mode and 122 detected in negative ion mode. The identified compounds include 110 flavonoids, 62 polyphenols, 125 amino acids, and 68 nucleic acids (Fig 8).

**Fig 9:**
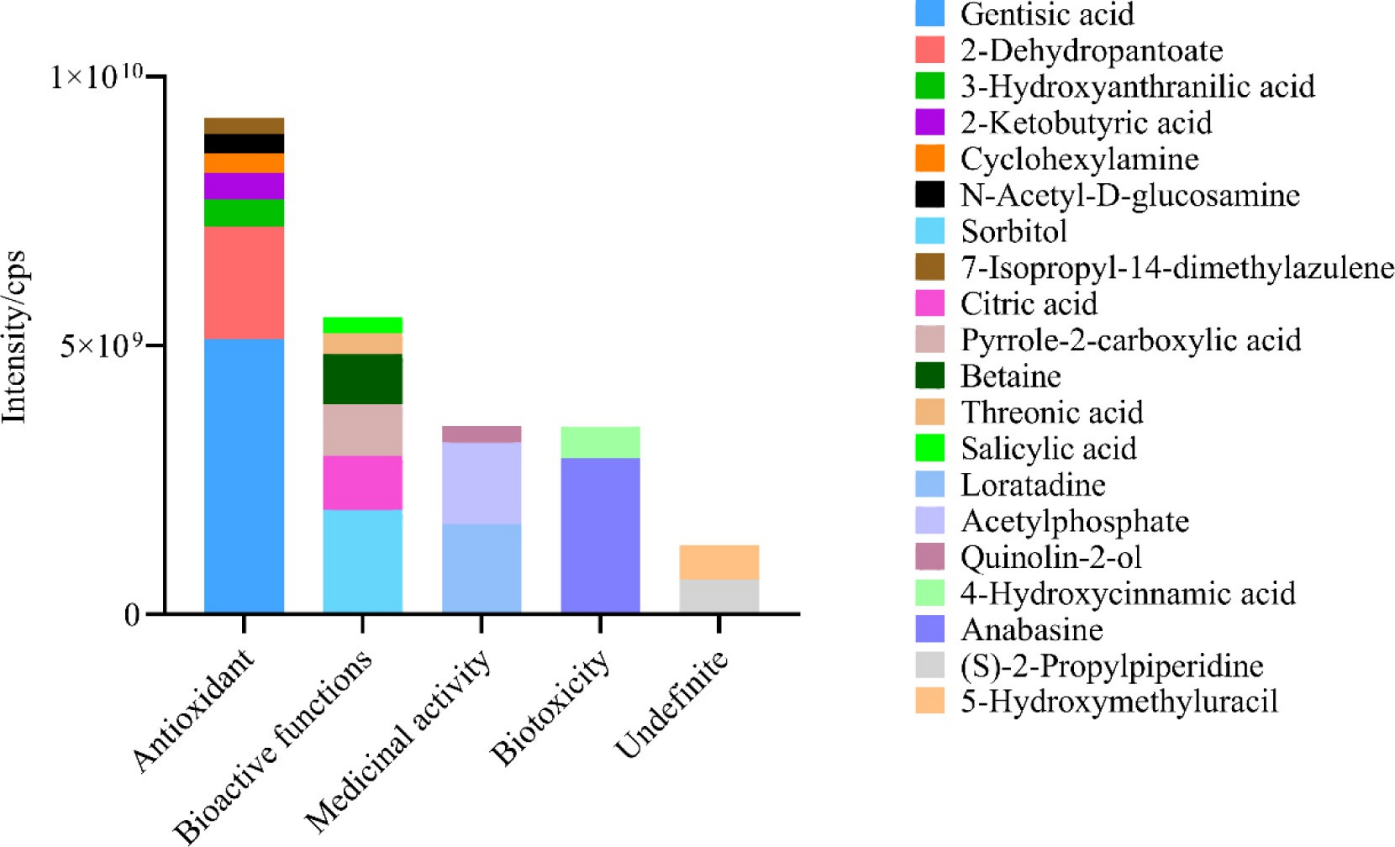
Types and contents of compounds in WNGH-AE (Top 20).

Among the top 20 compounds identified by WNGH-AE, they are classified according to functional classification in descending order of content as follows: Antioxidant compounds, Bioactive function compounds, Medicinal activity compounds, Biotoxicity compounds, and compounds with no special functions. The antioxidant functional compounds, ranked from highest to lowest content, are as follows: Gentisic acid, 2-Dehydropantoate, 3-Hydroxyanthranilic acid, 2-Ketobutyric acid, Cyclohexylamine, N-acetyl-D-glucosamine, and 7-Isopropyl-14-dimethylazulene. These compounds may represent the primary substances responsible for the antioxidant functions observed in WNGH-AE (Fig 9 and Table 3).

**Table 3:** LC–MS analysis of WNGH-AE.

Name	Formula	m/z	RT (s)	ppm	pos/neg	X (mean±SD)
Gentisic acid	C_7_H_6_O_4_	154.99	956.40	0.12	pos	5,116,121,774±3,717,780,087
Anabasine	C_10_H_14_N_2_	144.98	913.30	0.63	pos	2,895,173,015±353,436,351
2-Dehydropantoate	C_6_H_10_O_4_	146.06	322.10	0.15	pos	2,095,989,642±108,448,783
Sorbitol	C_6_H_14_O_6_	182.99	955.90	4.22	pos	1,934,965,639±1,015,802,151
Loratadine	C_10_H_17_N	152.14	885.10	1.31	pos	1,677,042,756±186,908,416
Acetyl phosphate	C_2_H_5_O_5_P	139.99	923.60	0.00	pos	1,514,082,546±349,671,037
Citric acid	C_6_H_8_O_7_	191.01	77.40	4.42	neg	1,009,387,543±38,573,331
Pyrrole-2-carboxylic acid	C_5_H_5_NO_2_	111.02	742.30	1.43	pos	958,563,699±507,959,379
Betaine	C_5_H_11_NO_2_	116.93	838.70	8.07	neg	929,921,074±615,409,418
(S) - 2-Propylpiperidine	C_8_H_17_N	128.14	755.60	2.93	pos	655,929,356±107,343,545
5-Hydroxymethyluracil	C_5_H_6_N_2_O_3_	141.96	958.30	2.58	pos	624,246,927±292,746,623
4-Hydroxycinnamic acid	C_9_H_8_O_3_	146.98	833.10	4.01	pos	589,833,847±684,949,662
3-Hydroxyanthranilic acid	C_7_H_7_NO_3_	154.05	212.60	2.44	pos	508,247,699±24,917,763
2-Ketobutyric acid	C_4_H_6_O_3_	102.03	344.70	2.60	pos	485,654,328±498,948,452
Threonic acid	C_4_H_8_O_5_	135.03	87.60	17.03	neg	398,433,716±13,716,305
Cyclohexylamine	C_6_H_13_N	100.11	535.70	7.23	pos	364,629,710±236,969,268
N-Acetyl-D-glucosamine	C_8_H_15_NO_6_	204.09	96.40	18.30	pos	358,245,214±7,575,713
Quinolin-2-ol	C_9_H_7_NO	146.06	283.50	3.59	pos	310,740,853±5,439,394
7-Isopropyl-1,4-dimethylazulene	C_15_H_18_	199.14	861.50	18.71	pos	296,875,483±230,967,101
Salicylic acid	C_7_H_6_O_3_	137.02	103.00	17.52	neg	295,649,849±14,331,218

Note: m/z: mass-to-charge ratio; RT: retention time; ppm: error between the detected molecular weight and the theoretical molecular weight in ppm; pos/neg: positive/negative; X (mean ± SD): mean ± standard deviation of the distribution of signals of the metabolite in a given type.

**Fig 10:**
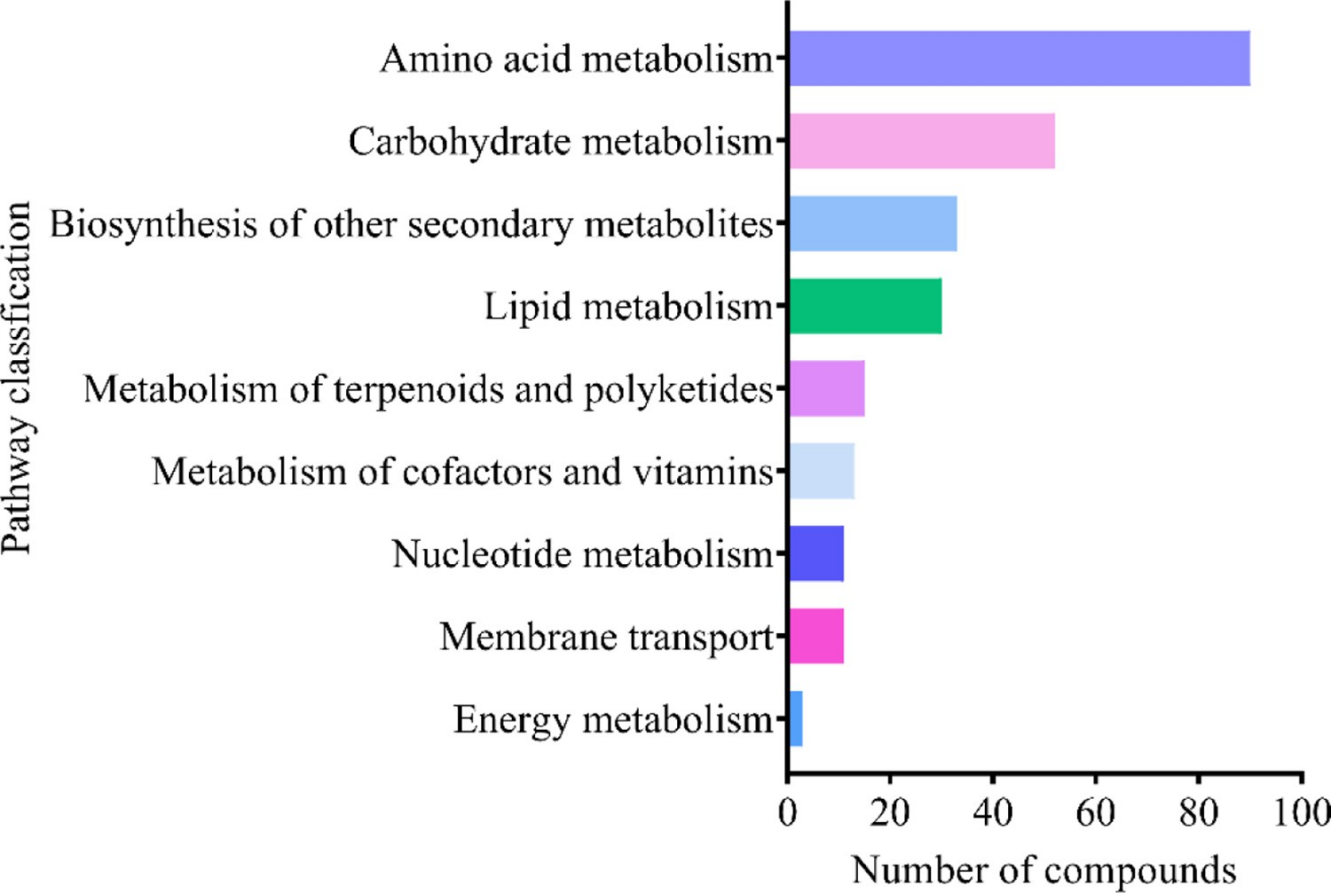
LC–MS analysis of WNGH-AE of number of KEGG pathway compounds.

Among the compounds identified by WNGH-AE, the order of compound content, categorized by their respective metabolic pathways, is as follows: Amino acid metabolism > Carbohydrate metabolism > Membrane transport > Lipid metabolism > Energy metabolism > Metabolism of terpenoids and polyketides > Nucleotide metabolism > Metabolism of cofactors and vitamins > Signal transduction; Metabolism of terpenoids and polyketides > Biosynthesis of other secondary metabolites. Notably, 86 compounds are associated with the amino acid metabolism pathway, while 52 compounds are linked to carbohydrate metabolism. This finding suggests that WNGH-AE predominantly influences the amino acid and carbohydrate metabolic pathways, as well as other metabolic functions in HepG2 cells, thereby contributing to the understanding of biological activity of WNGH-AE (Fig 10 and Table 4).

**Table 4:** LC–MS analysis of WNGH-AE of KEGG pathway.

KEGG pathway	Number of compounds	X (mean±SD)
Amino acid metabolism	90	21,485,545,126±4,686,113,640
Carbohydrate metabolism	52	10,870,702,645±1,519,682,435
Membrane transport	11	5,095,620,758±445,804,597
Lipid metabolism	30	3,992,960,114±364,991,773
Energy metabolism	3	3,507,664,627±114,141,675
Metabolism of terpenoids and polyketides	13	2,580,213,588±62,608,483
Nucleotide metabolism	11	2,428,750,073±86,213,806
Metabolism of cofactors and vitamins	13	2,288,596,169±83,325,008
Signal transduction; Metabolism of terpenoids and polyketides	2	110,296,701±7,210,505
Biosynthesis of other secondary metabolites	33	13,766,959,167±1,858,046,412

Note: X (mean ± SD): mean ± standard deviation of the distribution of signals of the metabolite in a given type.

## 4. Discussions

This study evaluated the biocompatibility of WNGH-AE on HepG2 cells with the MTT assay and LDH value measurement. The results indicated that WNGH-AE exhibited no toxic effects on HepG2 cells at experimental concentrations ranging from 0.0–0.4 mg/mL. It is noteworthy that WNGH is sometimes employed to investigate its effects on cancer cell proliferation, specifically its potential to inhibit or even induce cell death. Consequently, existing literature often demonstrates the toxic effects of WNGH extract on various cell types. For instance, a concentration of 9 mg/mL of WNGH methanol extract has been shown to inhibit the proliferation of nasopharyngeal carcinoma CNE-2 cells [35], while 7 mg/mL of WNGH ethanol extract similarly inhibits the proliferation of breast cancer MCF-7 cells [36]. These findings align with our research outcomes, which reveal that higher concentrations (0.8–12.8 mg/mL) of WNGH-AE can inhibit HepG2 cell proliferation in a concentration-dependent manner. As observed with many anticancer agents, WNGH not only impedes cancer cell proliferation but also affects normal cells [37]. However, this study specifically emphasizes the use of WNGH-AE as a pre-protective agent aimed at alleviating oxidative stress and inflammation within cells. Based on our findings, we recommend stringent concentration control when utilizing WNGH-AE as an antioxidant and anti-inflammatory agent to ensure its biological safety.

To evaluate the antioxidant capacity of WNGH, we utilized t-BHP to induce oxidative stress and inflammation in HepG2 cells, thereby constructing a pathological model. This model was employed to assess the antioxidant and anti-inflammatory properties of WNGH-AE. Our findings revealed that treatment with WNGH-AE led to the clearance of reactive oxygen species (ROS) and a reduction in DNA damage, as indicated by decreased levels of 8-OHdG. Furthermore, WNGH-AE effectively inhibited the activation of the NF-κB pathway and diminished the production of inflammatory cytokines TNF-α and IL-1β at a concentration of 0.1 mg/mL. Additionally, we observed that WNGH demonstrated the ability to scavenge DPPH and ·OH free radicals extracellularly (refer to Fig 7A and 7B). Notably, the water extract exhibited the most potent scavenging activity, with IC50 values of 0.027 and 0.96 mg/mL as shown in Table 1, suggesting that WNGH-AE holds significant potential for future applications.

Our preliminary analysis of the chemical composition of WNGH-AE revealed that it is rich in polysaccharide compounds and contains trace amounts of phenolic and ketone compounds. In addition, polysaccharide compounds exhibit the highest content in WNGH-AE, although there are relatively few types of these compounds. Additionally, WNGH-AE contains a small quantity of flavonoids and phenolic compounds, but encompasses a greater variety of types. This suggests that the antioxidant activity of WNGH-AE may be attributed to multiple substances, with polysaccharides potentially playing a major role. Furthermore, the substantial presence of amino acids indicates that WNGH-AE may possess several unrecognized bioactive functions. Notably, walnut husk polysaccharides and flavonoids have been shown to confer the ability of walnut husk to resist oxidative stress [38]. This functionality primarily relies on the inhibitory effects of walnut husk polysaccharides and flavonoids on the MAPK signaling transduction system, as well as their activation of the AKT pathway. The MAPK pathway [39], a key signal transduction system, is involved in regulating physiological processes such as cell growth, division, differentiation, and apoptosis. Inhibition of MAPK can effectively reduce the incidence of cell apoptosis [40]. Furthermore, the AKT pathway is closely associated with the oxidative stress response, and its activation can significantly mitigate cellular oxidative stress [41]. In this study, we found that WNGH-AE contains abundant polysaccharides and flavonoids, which may exert antioxidant effects by targeting MAPK and AKT in HepG2 cells. Additionally, the polysaccharide compounds within WNGH-AE can influence signaling pathways such as PI3K/AKT in cells. When the PI3K/AKT pathway is activated, it can modulate the intracellular NF-κB and Nrf2 pathways [42], thereby regulating oxidative stress and oxidative inflammation in cells. In summary, the antioxidant response of WNGH-AE may be achieved through the inhibition of the MAPK signaling pathway and the activation of the AKT pathway by polysaccharides and flavonoids. The anti-inflammatory effect may depend on the activation of the Nrf2 and PI3K/AKT pathways, along with the inhibition of the NF-κB pathway activation.

WNGH-AE exhibits significant potential for development within the healthcare sector. In this study, we made an intriguing discovery through LC-MS/MS analysis, identifying 403 compound molecules, which include 110 flavonoids, 62 polyphenols, 125 amino acids, and 68 nucleic acids, among others. Of the 20 most abundant organic molecules in WNGH-AE (as shown in Fig 9), 7 demonstrate antioxidant activity, 6 possess multiple biological activities, 3 are commonly utilized in drug development, and 2 exhibit biological toxicity. Notably, antioxidant-active substances such as gentian acid, 2-dehydropantothenic acid lactone, 3-hydroxyanthranilic acid, 2-butanone acid, and cyclohexylamine are present in high concentrations. In contrast, the levels of various bioactive substances, including citric acid and sorbitol, common food additives, are comparatively lower. Additionally, WNGH-AE contains well-known drug development candidates such as Loratadine and Acetyl phosphate (refer to Table 3). These findings suggest that WNGH-AE possesses a range of biological activities and holds promise for further development. The predominant compounds in WNGH-AE are polysaccharides. Given the safety of extraction agents, water is deemed the most suitable for extracting polysaccharides from walnut green husk for applications in food and medicine. For the extraction of other compounds from walnut green husk, alternative extraction agents should be employed.

WNGH-AE is a hydrophilic crude extract that exhibits cytotoxicity at elevated concentrations. This finding underscores the necessity of identifying and isolating its bioactive components in future development initiatives. In conclusion, our research demonstrates that WNGH-AE serves as a natural, high-quality antioxidant and anti-inflammatory agent with various potential biological functions. The antioxidant and anti-inflammatory properties of WNGH-AE are comparable to those of well-established natural antioxidants, such as green tea [43], curcumin supplementation [44], and resveratrol [45], thereby emphasizing the significant role of natural antioxidants in addressing diseases associated with oxidative stress and inflammation.

## 5. Conclusion

This study evaluated the toxicity, antioxidant, and anti-inflammatory effects of walnut green husk water extract on human HepG2 cells. The findings indicate that walnut green husk exhibits a certain level of toxicity to HepG2 cells, with a recommended concentration not exceeding 0.4 mg/mL. Furthermore, within the safe concentration range, walnut green husk water extract demonstrates excellent antioxidant and anti-inflammatory effects, significantly surpassing those of vitamin C. Analysis of the active compounds in the walnut green husk water extract revealed a substantial presence of polysaccharides and various bioactive substances, suggesting the potential for additional, yet undeveloped, functions. However, this study did not extract these compounds. Future research should focus on purifying and extracting effective bioactive substances from WNGH-AE and assessing their effects on AKT, MAPK targets, NF-κB, PI3K/AKT, and Nrf2 signaling pathways.

## Supporting information

S1 File LC MS method and results. (DOCX)

S2 File LC MS results. (XLSX)

S3 File Results of HepG2 cell. (XLS)

S4 File Results of chemical test. (XLSX)

S1 Graphical abstract (DOCX)

## Abbreviations

DPPH: 1,1-Diphenyl-2-picrylhydrazyl radical 2,2-Diphenyl-1-(2,4,6-trinitrophenyl) hydrazyl
HepG2: Human liver cancer cells
IL-1β: Interleukin-1β
LC-MS: Liquid Chromatograph Mass Spectrometer
LDH: Lactate dehydrogenase
MTT: 3-(3,5-dimethylthiazol-2-yl)-2,5-diphenyl-terazolium bromide assay
NADPH oxidase: Nicotinamide Adenine Dinucleotide Phosphate Oxidase
NF-κB: Nuclear factor κ-B
OH: Hydroxyl radical
ROS: Reactive oxygen species
t-BHP: Tert-butyl hydroperoxide
TNF-α: Tumor necrosis factor-α
VC: Vitamin C
WNGH: Walnut green husk
WNGH-AE: Walnut green husk aqueous extract
8-OHdG: 8-Hydroxy-2’-deoxyguanosine
