# Supplementary material for: Antioxidant and anti-inflammatory function of walnut green husk aqueous extract (WNGH-AE) on human hepatocellular carcinoma cells (HepG2) treated with t-BHP
Source: PLoS One. 2025 Jan 27;20(1):e0318005. doi: 10.1371/journal.pone.0318005 (PMC11771903; doi:10.1371/journal.pone.0318005)
Supplement: S1 File — (DOCX) [file pone.0318005.s001.docx]

**LC-MS analysis**

The freeze-dried WNGH-AE material was sent to Suzhou Panomics Biomedical Technology Co. for LC-MS analysis. Dissolve the samples in water, adjust the concentration to 1 g/mL, centrifuge at 12,000 rpm at 4 °C for 10 min, and filter the supernatant with a 0.22 µm filter membrane to obtain the solution to be measured. For chromatographic detection of WNGH-AE*,* an Ultra-High Performance Liquid Chromatography (UPLC) (ACQUITY, Waters, Milford, MA, USA) and Thermo Q Exactive (Thermo Fisher Scientific, USA) were used for mass spectrometry. The ion source was electrospray ionization (ESI). Chromatographic and mass spectrometry were performed in positive and negative ion modes, respectively.

Chromatographic conditions: ACQUITY UPLC® HSS T3 column (2.1×150 mm, 1.8 µm) (Waters, Milford, MA, USA), flow rate of 0.25 mL/min, column temperature of 40 ℃, injection volume of 2 μL. In positive ionization mode, the mobile phases consists of 0.1% formic acid-acetonitrile (C) and 0.1% formic acid-water (D), and the elution gradient is as follows: 0~1 min, 2% C; 1~9 min, 2%~50% C; 9~12 min, 50%~98% C; 12~13.5 min, at 98% C; 13.5~14 min, 98%~2% C; 14~20 min, 2% C. In the negative ionization mode, the mobile phases were acetonitrile (A) and 5 mmol/L ammonium formate aqueous solution (B) gradient elution, the elution program was: 0-1 min, 2% A; 1-9 min, 2%-50% A; 9-12 min, 50%-98% A; 12-13.5 min, 98% A; 13.5-14 min, 98%-2% A; and 14-17 min, 2% A^S1^.

The operating parameters of the mass spectrometry positive ion spray voltage of 3.50 kV, negative ion spray voltage of -2.50 kV, sheath gas of 30 Arb, auxiliary gas of 10 Arb, capillary temperature of 325 ℃, the primary full scan resolution of 70,000, and ion scan range of m/z 81~1000, and the HCD was used to perform the secondary fission, the collision voltage is 30% and the secondary resolution was 17500, The first 10 ions of the acquired signal were fragmented and unnecessary MS/MS information was removed by dynamic exclusion^S2^.

**Supplementary table 1:** LC-MS analysis of WNGH-AE

| Name | Formula | m/z | RT (s) | ppm | pos/neg | **X-1** | **X-2** | **X-3** |
| --- | --- | --- | --- | --- | --- | --- | --- | --- |
| Gentisic acid | C7H6O4 | 154.99 | 956.40 | 0.12 | pos | 9399271991.04 | 2723833807.11 | 3225259523.70 |
| Anabasine | C10H14N2 | 144.98 | 913.30 | 0.63 | pos | 3125330265.86 | 3071964196.18 | 2488224582.14 |
| 2-Dehydropantoate | C6H10O4 | 146.06 | 322.10 | 0.15 | pos | 1971857676.39 | 2143752486.09 | 2172358763.97 |
| Sorbitol | C6H14O6 | 182.99 | 955.90 | 4.22 | pos | 2356188722.70 | 2672395416.58 | 776312777.18 |
| Loratadine | C10H17N | 152.14 | 885.10 | 1.31 | pos | 1845803456.34 | 1709172601.15 | 1476152209.51 |
| Acetylphosphate | C2H5O5P | 139.99 | 923.60 | 0.00 | pos | 1915659060.38 | 1349654450.73 | 1276934127.10 |
| Citric acid | C6H8O7 | 191.02 | 77.40 | 4.42 | neg | 1045677617.02 | 1013607091.53 | 968877918.92 |
| Pyrrole-2-carboxylic acid | C5H5NO2 | 111.02 | 742.30 | 1.43 | pos | 540843695.21 | 810835381.46 | 1524012019.78 |
| Betaine | C5H11NO2 | 116.93 | 838.70 | 8.07 | neg | 1268335378.13 | 1301856759.60 | 219571083.94 |
| (S)-2-Propylpiperidine | C8H17N | 128.14 | 755.60 | 2.93 | pos | 765593689.70 | 651126551.05 | 551067826.38 |
| 5-Hydroxymethyluracil | C5H6N2O3 | 141.96 | 958.30 | 2.58 | pos | 934271676.47 | 352555178.41 | 585913927.13 |
| 4-Hydroxycinnamic acid | C9H8O3 | 146.98 | 833.10 | 4.01 | pos | 169245717.51 | 1380201405.03 | 220054417.70 |
| 3-Hydroxyanthranilic acid | C7H7NO3 | 154.05 | 212.60 | 2.44 | pos | 481740466.78 | 511810087.81 | 531192542.09 |
| 2-Ketobutyric acid | C4H6O3 | 102.03 | 344.70 | 2.60 | pos | 87015976.59 | 1045202813.86 | 324744193.33 |
| Threonic acid | C4H8O5 | 135.03 | 87.60 | 17.03 | neg | 390927007.11 | 414264901.44 | 390109238.28 |
| Cyclohexylamine | C6H13N | 100.11 | 535.70 | 7.23 | pos | 407930401.59 | 108995985.03 | 576962743.60 |
| N-Acetyl-D-glucosamine | C8H15NO6 | 204.09 | 96.40 | 18.30 | pos | 349517812.48 | 362093380.30 | 363124449.97 |
| Quinolin-2-ol | C9H7NO | 146.06 | 283.50 | 3.59 | pos | 305077258.92 | 311221100.13 | 315924199.62 |
| 7-Isopropyl-1,4-dimethylazulene | C15H18 | 199.14 | 861.50 | 18.71 | pos | 286311772.46 | 532943187.32 | 71371490.07 |
| Salicylic acid | C7H6O3 | 137.02 | 103.00 | 17.52 | neg | 312198099.67 | 287356418.71 | 287395029.17 |
| Guanidinosuccinic acid | C5H9N3O4 | 174.95 | 58.40 | 9.08 | neg | 462938888.84 | 322387137.99 | 64903663.94 |
| 2'-Deoxyguanosine | C10H13N5O4 | 266.94 | 925.90 | 1.06 | pos | 248762404.21 | 245821040.35 | 261686491.03 |
| 6-Hydroxynicotinic acid | C6H5NO3 | 139.12 | 774.50 | 1.88 | pos | 290693692.48 | 177124739.38 | 257481922.97 |
| (2S,5S)-trans-Carboxymethylproline | C7H11NO4 | 174.08 | 98.50 | 0.74 | pos | 222001330.12 | 240147169.30 | 230051322.41 |
| Caffeic acid | C9H8O4 | 163.04 | 352.50 | 18.25 | pos | 218261545.51 | 199557381.94 | 265983837.14 |
| Succinic acid semialdehyde | C4H6O3 | 102.03 | 718.00 | 3.06 | pos | 341014413.98 | 278905297.55 | 34913934.97 |
| D-Glucuronic Acid | C6H10O7 | 193.03 | 86.90 | 5.70 | neg | 214171116.56 | 248020231.20 | 191328205.77 |
| (1H-Indol-3-yl)-N-methylmethanamine | C10H12N2 | 160.11 | 195.10 | 1.20 | pos | 211468938.81 | 205688199.69 | 221841244.79 |
| L-Ribulose | C5H10O5 | 149.04 | 91.20 | 11.41 | neg | 202325414.95 | 201779104.05 | 204830396.40 |
| 2-Methyl-3-oxopropanoic acid | C4H6O3 | 102.03 | 738.20 | 2.62 | pos | 246038854.19 | 190986409.85 | 125092247.45 |
| L-Carnitine | C7H15NO3 | 162.11 | 94.30 | 2.00 | pos | 177655262.66 | 174401406.69 | 189157397.73 |
| trans-Aconitate | C6H6O6 | 157.01 | 98.60 | 27.60 | pos | 173404713.13 | 179232234.58 | 181880834.61 |
| Fumaric acid | C4H4O4 | 115.00 | 80.50 | 7.65 | neg | 167347849.01 | 160672900.16 | 173222884.93 |
| Phthalic acid | C8H6O4 | 149.02 | 605.20 | 17.68 | pos | 183274111.84 | 53745850.55 | 264103099.04 |
| Nicotinic acid | C6H5NO2 | 124.09 | 33.80 | 7.02 | pos | 143015849.56 | 149075404.21 | 168945149.13 |
| 2-Oxo-4-methylthiobutanoic acid | C5H8O3S | 149.02 | 195.20 | 8.08 | pos | 292479068.33 | 79339543.56 | 88690108.52 |
| Coumarin | C9H6O2 | 147.04 | 348.90 | 1.07 | pos | 139456892.43 | 147827091.89 | 149614123.98 |
| 11-Dehydrocorticosterone | C21H28O4 | 325.18 | 843.40 | 6.73 | neg | 95912631.72 | 107702662.30 | 225038847.17 |
| 1-Pyrroline-4-hydroxy-2-carboxylate | C5H7NO3 | 130.05 | 755.50 | 1.63 | pos | 255742688.72 | 34947974.70 | 130393495.64 |
| 2,3-Butanediol | C4H10O2S2 | 154.99 | 694.20 | 2.15 | pos | 167012881.32 | 193941686.55 | 48010092.28 |
| Triacetate lactone | C6H6O3 | 127.04 | 36.40 | 0.05 | pos | 111902332.37 | 114228337.27 | 163227866.07 |
| Procaine | C13H20N2O2 | 236.15 | 110.20 | 15.67 | pos | 117635925.29 | 124914059.20 | 122015254.97 |
| 4-Acetamidobutanoic acid | C6H11NO3 | 146.08 | 250.10 | 0.52 | pos | 111657824.19 | 113851499.88 | 115379734.92 |
| Pyroglutamic acid | C5H7NO3 | 130.05 | 585.30 | 0.01 | pos | 85276857.20 | 24688229.54 | 229392602.24 |
| Quinate | C7H12O6 | 191.05 | 84.50 | 3.22 | neg | 155242561.28 | 97551261.17 | 83021737.82 |
| Exemestane | C20H24O2 | 279.16 | 593.30 | 20.30 | pos | 90822616.25 | 76925119.61 | 165652195.86 |
| Catechol | C6H6O2 | 111.04 | 406.40 | 0.95 | pos | 92951569.04 | 111025671.92 | 128907246.60 |
| Phosphoglycolic acid | C2H5O6P | 156.99 | 739.00 | 15.44 | pos | 57691199.83 | 62138552.54 | 211083480.23 |
| Pyrroline hydroxycarboxylic acid | C5H7NO3 | 130.05 | 636.30 | 4.80 | pos | 119761425.81 | 27713342.65 | 183091469.14 |
| Dodecanoic acid | C12H24O2 | 199.97 | 962.40 | 7.34 | pos | 172753724.18 | 71780067.72 | 77196197.67 |
| 4-Guanidinobutanal | C5H11N3O | 130.09 | 818.60 | 5.92 | pos | 71999001.64 | 62023916.66 | 185534383.34 |
| Dehydroepiandrosterone | C19H28O2 | 288.29 | 673.90 | 0.67 | pos | 21056897.44 | 264822216.62 | 26911804.17 |
| Riboflavin | C17H20N4O6 | 377.15 | 373.80 | 0.86 | pos | 90810401.24 | 105814409.68 | 102353612.86 |
| Pelargonic acid | C9H18O2 | 158.99 | 691.60 | 0.81 | pos | 57717256.40 | 167863509.75 | 67137433.06 |
| 3-Indoleacetonitrile | C10H8N2 | 156.12 | 708.90 | 1.37 | pos | 72002797.36 | 116749590.02 | 101860422.71 |
| Lariciresinol | C20H24O6 | 361.17 | 453.50 | 4.92 | pos | 61684092.95 | 59173606.17 | 162764193.94 |
| L-Proline | C5H9NO2 | 116.07 | 665.30 | 0.28 | pos | 182580568.82 | 68272736.05 | 32508044.97 |
| (S)-1-Phenylethanol | C8H10O | 123.04 | 734.80 | 0.97 | pos | 92201038.36 | 73897035.59 | 116997455.14 |
| Glyceric acid | C3H6O4 | 105.02 | 87.60 | 5.17 | neg | 92015537.96 | 93982500.24 | 93420590.33 |
| Undecanoic acid | C11H22O2 | 186.96 | 65.80 | 0.23 | pos | 100120733.87 | 88022777.18 | 90402528.44 |
| Cyclopeptine | C17H16N2O2 | 280.10 | 110.50 | 0.78 | pos | 92900410.45 | 92686151.14 | 90846600.65 |
| Ciliatine | C2H8NO3P | 125.99 | 661.70 | 6.58 | pos | 198238921.86 | 17460092.36 | 58839638.54 |
| Argininosuccinic acid | C10H18N4O6 | 291.13 | 115.20 | 0.02 | pos | 86526835.52 | 84298491.51 | 91392259.00 |
| D-4-Hydroxy-2-oxoglutarate | C5H6O6 | 163.04 | 156.70 | 7.52 | pos | 161530645.54 | 45536570.36 | 55123584.16 |
| Hydroquinone | C6H6O2 | 110.02 | 492.00 | 0.98 | pos | 131647157.65 | 34262929.33 | 96248141.61 |
| 4-Oxoproline | C5H7NO3 | 130.05 | 977.40 | 3.66 | pos | 88437094.79 | 73187078.59 | 88232165.28 |
| D-Fructose | C6H12O6 | 181.01 | 721.20 | 3.63 | pos | 100949892.95 | 122127049.04 | 19863054.84 |
| Methyloxaloacetate | C5H6O5 | 147.03 | 171.30 | 0.48 | pos | 79069332.44 | 78243901.20 | 85373573.17 |
| Dibutyl phthalate | C16H22O4 | 279.16 | 747.70 | 13.88 | pos | 55856426.76 | 91422965.11 | 94893893.57 |
| Fructose 1,6-bisphosphate | C6H14O12P2 | 339.20 | 887.60 | 1.59 | neg | 98949698.80 | 96701478.04 | 43594590.77 |
| Pterin | C6H5N5O | 163.05 | 930.90 | 7.97 | pos | 39203020.11 | 30152263.46 | 158998802.64 |
| Dethiobiotin | C10H18N2O3 | 213.98 | 957.20 | 1.74 | pos | 25107236.06 | 82280215.26 | 120160060.90 |
| L-Malic acid | C4H6O5 | 133.05 | 97.70 | 1.60 | neg | 78937331.15 | 76339371.93 | 71404708.59 |
| Nornicotine | C9H12N2 | 146.96 | 809.70 | 4.65 | neg | 64190345.36 | 81035615.73 | 79597049.64 |
| 2-Keto-glutaramic acid | C5H7NO4 | 145.05 | 95.20 | 0.11 | pos | 67491873.32 | 74348286.12 | 82977923.86 |
| Acetoacetic acid | C4H6O3 | 102.03 | 146.90 | 27.44 | pos | 73225017.00 | 88231909.90 | 62391581.39 |
| (2E)-Decenoyl-ACP | C6H11NO2 | 130.09 | 759.80 | 1.19 | pos | 54427045.38 | 70501506.39 | 95593839.66 |
| Diisobutyl phthalate | C16H22O4 | 279.16 | 980.10 | 13.70 | pos | 69047862.24 | 71193193.57 | 80010851.85 |
| D-Mannose | C6H12O6 | 181.01 | 743.50 | 3.46 | pos | 48393711.63 | 153581021.10 | 15773893.25 |
| trans-Ferulic acid | C10H10O4 | 195.07 | 443.10 | 3.59 | pos | 65110307.59 | 69252751.33 | 75756143.88 |
| L-4-Hydroxyphenylglycine | C8H9NO3 | 167.01 | 38.80 | 1.33 | pos | 135479321.73 | 56795080.71 | 16999353.90 |
| (R)-5,6-Dihydrothymine | C5H8N2O2 | 128.07 | 246.90 | 4.99 | pos | 66771529.63 | 68502900.62 | 69314625.67 |
| Diethyl phthalate | C12H14O4 | 205.09 | 937.10 | 26.50 | pos | 134699122.58 | 39695025.62 | 28624879.26 |
| Kynurenic acid | C10H7NO3 | 190.05 | 345.70 | 0.40 | pos | 65513556.16 | 67498086.82 | 69811305.90 |
| 5-Hydroxypyrazinamide | C5H5N3O | 123.04 | 438.00 | 17.30 | pos | 88352426.08 | 22435151.92 | 90543651.60 |
| Chlorogenic acid | C16H18O9 | 355.10 | 352.50 | 0.91 | pos | 63381097.99 | 63725008.96 | 66986531.71 |
| 1-Hydroxy-2-naphthoate | C11H8O3 | 170.98 | 946.50 | 7.08 | pos | 81755621.32 | 49830047.03 | 61956503.25 |
| 4-Hydroxy-3-methoxy-benzaldehyde | C8H8O3 | 153.05 | 456.60 | 2.12 | pos | 65474261.19 | 60561064.52 | 63991305.75 |
| 3-(2-Hydroxyphenyl)propanoic acid | C9H10O3 | 146.96 | 640.00 | 0.14 | neg | 79787221.71 | 77311123.21 | 32442684.40 |
| 2-Oxo-4-phenylbutyric acid | C10H10O3 | 179.07 | 514.30 | 2.93 | pos | 58673077.67 | 62945844.57 | 64599399.00 |
| Gallic acid | C7H6O5 | 169.01 | 95.30 | 15.38 | neg | 55714598.33 | 63213103.85 | 58068445.14 |
| N-Carbamoylputrescine | C5H13N3O | 132.10 | 550.40 | 1.66 | pos | 94978867.87 | 18956475.44 | 62497436.03 |
| 2-Hydroxy-3-oxoadipate | C6H8O6 | 177.06 | 362.90 | 8.96 | pos | 50129349.30 | 52262982.23 | 72791799.15 |
| Sweroside | C16H22O9 | 339.10 | 368.30 | 10.00 | neg | 58939570.48 | 52511669.94 | 63205023.77 |
| Isoquercitrin | C21H20O12 | 465.10 | 428.40 | 1.45 | pos | 49086820.60 | 51601950.05 | 69596172.61 |
| Uracil | C4H4N2O2 | 113.03 | 141.70 | 0.10 | pos | 53541896.85 | 55657367.13 | 58487343.30 |
| L-Asparagine | C4H8N2O3 | 132.10 | 788.00 | 7.12 | pos | 47203078.26 | 63398750.95 | 51032210.18 |
| Sedoheptulose | C7H14O7 | 209.06 | 90.60 | 11.00 | neg | 53632380.65 | 53804272.86 | 52741634.00 |
| Demethylcitalopram | C19H19FN2O | 311.15 | 470.60 | 27.24 | pos | 49847880.50 | 56008111.35 | 48828590.96 |
| 2-Keto-6-aminocaproate | C6H11NO3 | 145.07 | 377.10 | 17.23 | pos | 48424711.15 | 51670256.21 | 53624347.09 |
| Pelletierine | C8H15NO | 142.12 | 830.30 | 5.09 | pos | 78615898.47 | 16669172.47 | 56743143.49 |
| N2-Succinyl-L-arginine | C10H18N4O5 | 275.14 | 160.60 | 0.45 | pos | 48829284.23 | 48451058.32 | 51386825.09 |
| Dihydrouracil | C4H6N2O2 | 112.98 | 880.00 | 5.64 | neg | 43395743.45 | 47798609.80 | 55478567.73 |
| L-Rhamnofuranose | C6H12O5 | 164.07 | 565.80 | 4.88 | pos | 24894277.10 | 70104331.47 | 51043431.36 |
| Citrulline | C6H13N3O3 | 158.09 | 193.60 | 29.31 | pos | 50051093.77 | 47421983.85 | 47371688.51 |
| 6-Methylmercaptopurine | C6H6N4S | 166.03 | 168.20 | 29.51 | pos | 40220918.87 | 50340315.52 | 52813606.90 |
| O-Acetylserine | C5H9NO4 | 148.06 | 123.80 | 0.84 | pos | 47461406.44 | 49177789.41 | 46224935.68 |
| Xanthine | C5H4N4O2 | 153.04 | 207.30 | 0.33 | pos | 44712798.69 | 46557645.02 | 50954019.74 |
| 3-Hydroxyphenylacetic acid | C8H8O3 | 153.00 | 934.10 | 1.33 | pos | 83562843.97 | 26063614.23 | 31908874.62 |
| Aspirin | C9H8O4 | 163.04 | 794.20 | 14.93 | pos | 54411466.21 | 20228450.76 | 63325652.64 |
| 6-Phosphogluconic acid | C6H13O10P | 277.03 | 95.00 | 1.36 | pos | 40007936.69 | 53375863.65 | 41239666.10 |
| Mannitol | C6H14O6 | 183.08 | 931.90 | 29.36 | pos | 38200296.36 | 44918719.35 | 48240747.28 |
| (2Z,4S,5R)-2-Amino-4,5,6-trihydroxyhex-2-enoate | C6H11NO5 | 160.06 | 138.70 | 28.33 | pos | 42514106.87 | 44185855.67 | 41866515.52 |
| L-Threo-2-pentulose | C5H10O5 | 131.03 | 93.70 | 9.23 | neg | 47773248.42 | 48184982.37 | 32515013.37 |
| 4-Pyridoxic acid | C8H9NO4 | 184.06 | 253.30 | 2.30 | pos | 39274790.67 | 39514727.64 | 44699390.03 |
| 3-Aminopentanedioate | C5H9NO4 | 128.03 | 944.10 | 19.60 | neg | 27561665.60 | 64160771.03 | 31723500.96 |
| Adenosine | C10H13N5O4 | 268.10 | 238.40 | 1.12 | pos | 40655486.56 | 40728488.53 | 41843417.75 |
| Tetrahydrodipicolinate | C7H9NO4 | 172.06 | 112.10 | 0.08 | pos | 37514318.33 | 40734148.51 | 42457992.69 |
| 10-Hydroxydecanoic acid | C10H20O3 | 171.14 | 677.00 | 25.32 | pos | 39621986.89 | 41205372.54 | 39607141.93 |
| beta-Alanyl-L-arginine | C9H19N5O3 | 245.15 | 265.60 | 0.04 | pos | 34277381.06 | 39308705.78 | 39948862.45 |
| Quinolinic acid | C7H5NO4 | 167.01 | 330.30 | 1.87 | pos | 36854993.21 | 38302511.23 | 37157738.49 |
| Pentaporphyrin I | C19H21NO4 | 327.16 | 501.20 | 0.11 | pos | 29601725.81 | 33926307.43 | 48158256.56 |
| 3D-3,5_4-Trihydroxycyclohexane-1,2-dione | C6H8O5 | 161.06 | 476.90 | 7.11 | pos | 30910700.28 | 30596190.17 | 48636948.88 |
| Mechlorethamine | C5H11Cl2N | 156.03 | 139.20 | 28.20 | pos | 35609849.27 | 35643811.95 | 38812405.54 |
| 3,4-Dihydroxyphthalate | C8H6O6 | 197.01 | 115.40 | 8.75 | neg | 36033296.02 | 36602737.86 | 37183603.14 |
| Beta-D-Glucose 6-phosphate | C6H13O9P | 261.06 | 97.00 | 0.18 | pos | 44229108.17 | 12506070.75 | 51926669.86 |
| 5-Aminopentanoic acid | C5H11NO2 | 118.09 | 231.00 | 3.57 | pos | 89220592.69 | 8323487.35 | 8692790.86 |
| Pantothenic acid | C9H17NO5 | 218.10 | 197.40 | 3.13 | neg | 34254105.70 | 35556254.91 | 34708613.01 |
| N(6)-Methyllysine | C7H16N2O2 | 161.13 | 441.20 | 0.42 | pos | 33807158.17 | 34588628.45 | 35605008.24 |
| N6-Acetyl-N6-hydroxy-L-lysine | C8H16N2O4 | 204.12 | 256.80 | 0.02 | pos | 32829356.13 | 33961933.38 | 36614754.25 |
| cis-Aconitic acid | C6H6O6 | 157.01 | 171.20 | 28.24 | pos | 33294310.93 | 35210865.00 | 34736234.61 |
| 3-Dehydro-scyllo-inosose | C6H8O6 | 177.06 | 514.80 | 23.36 | pos | 35568067.52 | 29407422.86 | 37993318.64 |
| L-2-Hydroxyglutaric acid | C5H8O5 | 146.96 | 792.90 | 4.65 | neg | 41340728.41 | 33545136.63 | 27787975.91 |
| Nicotinate D-ribonucleoside | C11H14NO6 | 256.08 | 111.50 | 1.26 | pos | 42685334.11 | 12633217.95 | 46115700.70 |
| 3-(3,4-Dihydroxy-5-methoxy)-2-propenoic acid | C10H10O5 | 193.05 | 465.80 | 18.31 | pos | 32443397.14 | 32071756.74 | 36451780.71 |
| Ureidosuccinic acid | C5H8N2O5 | 177.06 | 427.30 | 26.12 | pos | 45909140.08 | 42534254.77 | 11613417.96 |
| cis-4-Hydroxy-D-proline | C5H9NO3 | 131.97 | 64.80 | 1.89 | pos | 40243131.63 | 44072008.82 | 10709941.36 |
| 2-Hydroxy-6-pentadecylbenzoic acid | C22H36O3 | 348.27 | 622.70 | 20.67 | pos | 29092706.53 | 32737295.55 | 32825631.41 |
| Urocanic acid | C6H6N2O2 | 139.05 | 141.00 | 0.63 | pos | 30113708.47 | 29819130.39 | 33357167.43 |
| Perillyl aldehyde | C10H14O | 151.11 | 612.70 | 1.08 | pos | 27047333.16 | 32182928.30 | 32427686.37 |
| Quercetin | C15H10O7 | 303.05 | 557.10 | 3.22 | pos | 29449113.23 | 31641385.08 | 30497870.78 |
| Sebacic acid | C10H18O4 | 185.11 | 620.70 | 0.06 | pos | 32230075.54 | 46529980.47 | 12271179.07 |
| Gabapentin | C9H17NO2 | 172.13 | 645.40 | 4.31 | pos | 11876778.48 | 11538986.07 | 67427901.78 |
| Arachidic acid | C20H40O2 | 311.17 | 854.30 | 1.27 | neg | 9634028.24 | 62157992.02 | 16285176.14 |
| 3-Hydroxypicolinic acid | C6H5NO3 | 140.03 | 251.80 | 0.06 | pos | 28234761.88 | 29711321.84 | 29732486.55 |
| D-Arabitol | C5H12O5 | 153.08 | 95.60 | 1.02 | pos | 28598017.82 | 28080457.80 | 30554732.12 |
| Securinine | C13H15NO2 | 217.11 | 288.00 | 23.85 | pos | 29346171.50 | 28749650.27 | 28105395.68 |
| Benzene-1,2,4-triol | C6H6O3 | 126.03 | 962.30 | 14.28 | pos | 37807230.61 | 7494915.91 | 40195349.87 |
| Malonate | C3H4O4 | 103.00 | 85.30 | 4.51 | neg | 27014903.84 | 29506661.33 | 28608680.86 |
| Epsilon-caprolactam | C6H11NO | 114.09 | 707.20 | 0.01 | pos | 25171593.39 | 26739857.64 | 32728829.63 |
| Gluconic acid | C6H12O7 | 197.07 | 95.60 | 3.17 | pos | 28761523.11 | 27986489.17 | 27829801.95 |
| Guanine | C5H5N5O | 152.06 | 131.20 | 0.82 | pos | 26359308.35 | 27254767.49 | 29830903.04 |
| 2,6-Diethylaniline | C10H15N | 149.12 | 784.70 | 24.14 | pos | 7156690.27 | 7990712.46 | 66580516.00 |
| Hippuric acid | C9H9NO3 | 180.07 | 140.90 | 1.53 | pos | 26278196.58 | 26547935.20 | 26623372.99 |
| 2-Keto-6-acetamidocaproate | C8H13NO4 | 188.09 | 182.90 | 1.47 | pos | 25265485.28 | 27993724.37 | 25793035.67 |
| gamma-Aminobutyric acid | C4H9NO2 | 104.07 | 92.30 | 0.95 | pos | 26930254.47 | 25792561.53 | 23891215.27 |
| Aminohydroquinone | C6H7NO2 | 126.05 | 185.10 | 1.40 | pos | 8404122.17 | 27961230.98 | 39078616.34 |
| 9,10-EOT | C18H28O3 | 293.21 | 667.80 | 1.11 | pos | 23249120.47 | 25372739.44 | 24424111.22 |
| 2',6'-Dihydroxy-4'-methoxyacetophenone | C9H10O4 | 183.07 | 462.70 | 0.96 | pos | 23563969.05 | 23067083.99 | 25763480.61 |
| Ketoleucine | C6H10O3 | 130.01 | 934.00 | 2.69 | pos | 36036037.99 | 10146564.76 | 26095516.42 |
| D-Xylonate | C5H10O6 | 147.03 | 109.00 | 7.55 | neg | 23829713.85 | 24009983.97 | 23952846.64 |
| Stearic acid | C18H36O2 | 283.26 | 831.50 | 6.09 | neg | 23544800.73 | 24168374.30 | 22407485.80 |
| Dolichotheline | C10H17N3O | 195.12 | 288.00 | 6.55 | pos | 23830245.34 | 22226412.73 | 23626312.60 |
| L-Kynurenine | C10H12N2O3 | 209.15 | 760.80 | 3.48 | pos | 19549666.20 | 22643656.92 | 24975436.36 |
| Perindopril | C19H32N2O5 | 367.23 | 819.60 | 15.18 | neg | 22198264.29 | 22219125.97 | 22606332.39 |
| 5-Methyl-2-furancarboxaldehyde | C6H6O2 | 111.04 | 103.90 | 0.06 | pos | 4550008.82 | 4729933.79 | 55642311.53 |
| 5-Aminolevulinic acid | C5H9NO3 | 132.07 | 195.80 | 3.03 | pos | 21127988.42 | 21969731.41 | 21671297.49 |
| L-Phenylalanine | C9H11NO2 | 166.09 | 281.70 | 2.54 | pos | 22287248.41 | 21142776.55 | 19715222.71 |
| Beta-Leucine | C6H13NO2 | 130.09 | 730.20 | 15.56 | neg | 30730127.19 | 24954512.75 | 6700305.00 |
| Shikimic acid | C7H10O5 | 173.04 | 84.50 | 4.96 | neg | 21675716.39 | 25433392.95 | 14956453.39 |
| Aesculetin | C9H6O4 | 177.02 | 385.70 | 11.86 | neg | 22722665.07 | 20793493.46 | 18161922.14 |
| Neocnidilide | C12H18O2 | 195.14 | 623.10 | 2.93 | pos | 18606262.48 | 21192342.18 | 21157009.13 |
| 4-Hydroxycinnamoylmethane | C10H10O2 | 162.08 | 161.00 | 1.97 | pos | 7661666.04 | 19083911.20 | 32525026.92 |
| L-Valine | C5H11NO2 | 118.09 | 253.50 | 1.05 | pos | 6282531.72 | 23817520.05 | 28284382.42 |
| Pipecolic acid | C6H11NO2 | 130.09 | 174.30 | 13.97 | pos | 20131978.81 | 19482835.27 | 17502447.04 |
| Thymine | C5H6N2O2 | 127.05 | 275.50 | 0.61 | pos | 19627472.68 | 16546517.28 | 20588394.17 |
| 4,5-Dihydroorotic acid | C5H6N2O4 | 158.15 | 180.60 | 4.85 | pos | 15503412.18 | 20962869.97 | 19811363.35 |
| L-Isoleucine | C6H13NO2 | 130.09 | 620.80 | 7.01 | neg | 14738923.79 | 32582244.72 | 8509529.40 |
| Cytidine | C9H13N3O5 | 244.09 | 125.40 | 0.38 | pos | 18178862.27 | 18107420.75 | 19324586.60 |
| Phenylacetic acid | C8H8O2 | 137.13 | 115.60 | 8.50 | pos | 36100335.02 | 9415435.50 | 9841516.37 |
| N-Acetylglutamic acid | C7H11NO5 | 189.09 | 140.40 | 3.00 | pos | 17545888.63 | 18158764.16 | 19462967.03 |
| 3-[(1-Carboxyvinyl)oxy]benzoate | C10H8O5 | 191.03 | 430.80 | 23.73 | pos | 18323398.70 | 17758529.23 | 18415901.59 |
| N-Vinyl-2-pyrrolidone | C6H9NO | 112.08 | 882.80 | 1.11 | pos | 16590938.85 | 24678183.89 | 13068139.38 |
| Gulonic acid | C6H12O7 | 177.04 | 810.80 | 11.92 | neg | 10660994.55 | 31533530.71 | 12109395.76 |
| 12-Hydroxydodecanoic acid | C12H24O3 | 216.20 | 579.10 | 3.28 | pos | 16703715.38 | 18077527.41 | 18556776.40 |
| 1,2-Epoxy-p-menth-8-ene | C10H16O | 153.13 | 464.20 | 2.61 | pos | 17777328.90 | 15824081.08 | 18921756.36 |
| L-Cysteine | C3H7NO2S | 122.06 | 148.00 | 26.39 | pos | 19007315.30 | 16471712.15 | 16913078.68 |
| Pentyl octanoate | C18H30O3 | 277.22 | 623.00 | 16.72 | pos | 17846920.17 | 17319653.61 | 17155362.49 |
| Myristic acid | C14H28O2 | 229.18 | 676.70 | 4.84 | pos | 16294589.89 | 15617024.97 | 18587637.87 |
| 6-Methoxymellein | C11H12O4 | 209.08 | 586.60 | 2.03 | pos | 16945452.52 | 15194031.48 | 18155201.78 |
| 9-Riburonosyladenine | C10H11N5O5 | 281.10 | 301.80 | 2.21 | pos | 4969441.22 | 21580554.51 | 23105036.60 |
| Myristicin | C11H12O3 | 193.09 | 482.80 | 1.22 | pos | 14728853.19 | 16249239.77 | 18416070.60 |
| Jasmone | C11H16O | 165.13 | 722.40 | 3.17 | pos | 16108705.60 | 16217981.70 | 16850478.32 |
| (E)-3-(4-Hydroxyphenyl)-2-propenal | C9H8O2 | 149.06 | 367.60 | 2.65 | pos | 16233481.68 | 16846552.82 | 16092045.80 |
| Estragole | C10H12O | 149.10 | 533.30 | 0.05 | pos | 11927720.48 | 13081619.83 | 23281701.04 |
| Methyl beta-D-galactoside | C7H14O6 | 176.97 | 218.90 | 6.07 | pos | 16047738.79 | 15434010.02 | 16688614.19 |
| Carnosine | C9H14N4O3 | 209.09 | 248.80 | 24.23 | pos | 15777454.68 | 15764257.85 | 16551504.64 |
| D-Ribose | C5H10O5 | 151.04 | 38.10 | 4.96 | pos | 11925510.08 | 11077020.41 | 24879041.74 |
| 9(S)-HPOT | C18H30O4 | 293.21 | 710.60 | 0.51 | pos | 14449972.67 | 16274578.22 | 17148932.58 |
| Vanillylmandelic acid | C9H10O5 | 198.19 | 614.20 | 2.18 | pos | 14748032.11 | 15833385.57 | 17201296.64 |
| Vanylglycol | C9H12O4 | 165.05 | 253.70 | 11.57 | neg | 19723231.87 | 12262504.14 | 15673103.98 |
| Sinapoyl aldehyde | C11H12O4 | 209.08 | 510.50 | 0.50 | pos | 16157512.30 | 13995173.56 | 17040503.76 |
| 3-Methyl-L-tyrosine | C10H13NO3 | 195.10 | 527.90 | 3.57 | pos | 15111597.71 | 15187904.11 | 16375512.92 |
| Erucic acid | C22H42O2 | 338.34 | 806.30 | 2.57 | pos | 28565864.65 | 9470529.96 | 8606612.94 |
| 2,3-Dinor-8-iso prostaglandin F2alpha | C18H30O5 | 309.21 | 682.00 | 0.02 | pos | 15058679.10 | 14571214.00 | 16655248.56 |
| Phenylethylamine | C8H11N | 121.03 | 212.60 | 12.20 | neg | 26346374.05 | 3566305.50 | 16278006.04 |
| Phenyl acetate | C8H8O2 | 134.89 | 844.00 | 9.15 | neg | 13603345.55 | 25812380.09 | 6329469.31 |
| N-Acetyl-L-phenylalanine | C11H13NO3 | 208.10 | 283.50 | 2.29 | pos | 15585630.86 | 14731965.24 | 14944406.84 |
| L-Arginine | C6H14N4O2 | 174.11 | 158.00 | 1.72 | pos | 18927371.50 | 20389948.20 | 5393586.46 |
| trans-Cinnamate | C9H8O2 | 146.96 | 740.80 | 1.66 | neg | 14977260.79 | 14853203.36 | 14673970.70 |
| Enalaprilat | C18H24N2O5 | 349.18 | 904.70 | 16.81 | pos | 15092365.83 | 15466272.04 | 13251403.50 |
| 3-Hydroxybenzoic acid | C7H6O3 | 137.02 | 206.90 | 6.45 | neg | 18762468.85 | 7793331.66 | 17168744.82 |
| Ascorbate | C6H8O6 | 176.97 | 68.20 | 0.97 | pos | 14431540.19 | 7098502.79 | 22073882.73 |
| (1S,2R,4S)-(-)-Bornyl acetate | C12H20O2 | 179.14 | 750.20 | 24.75 | pos | 13408376.74 | 15771589.19 | 14101419.67 |
| 4-Guanidinobutanoic acid | C5H11N3O2 | 144.08 | 103.60 | 4.70 | neg | 15183924.35 | 12693489.44 | 14567266.31 |
| N6-Acetyl-L-lysine | C8H16N2O3 | 189.13 | 520.30 | 19.56 | pos | 20735169.46 | 10070022.42 | 10891485.41 |
| 3-Hydroxymethylglutaric acid | C6H10O5 | 145.05 | 952.40 | 27.81 | pos | 7860858.04 | 22693605.41 | 10889560.52 |
| Psoralidin | C20H16O5 | 337.11 | 105.30 | 12.23 | pos | 6501782.76 | 25997528.76 | 8512289.44 |
| Econazole | C18H15Cl3N2O | 381.02 | 539.00 | 24.61 | pos | 12753989.90 | 12312405.33 | 15372853.66 |
| 2-Hydroxy-1,4-naphthoquinone | C10H6O3 | 175.04 | 369.30 | 1.71 | pos | 12219235.15 | 13944256.16 | 13862167.38 |
| 3-Dehydroshikimate | C7H8O5 | 171.99 | 35.10 | 0.11 | pos | 10678782.26 | 12980006.52 | 15437065.94 |
| O-Acetyl-L-homoserine | C6H11NO4 | 162.08 | 189.80 | 0.90 | pos | 3818184.18 | 16879225.77 | 18254705.71 |

*: m/z: mass-to-charge ratio; RT: retention time; ppm: error between the detected molecular weight and the theoretical molecular weight in ppm; pos/neg: positive/negative; X (mean ± SD): mean ± standard deviation of the distribution of signals of the metabolite in a given type.

**References**

S1. Zelena, E., Dunn, W.B., Broadhurst, D., Francis-McIntyre, S., Carroll, K.M., Begley, P., O’Hagan, S., Knowles, J. D., Halsall, A., Wilson, I. D., Kell, D. B. Development of a Robust and Repeatable UPLC−MS Method for the Long-Term Metabolomic Study of Human Serum. *Analytical Chemistry* **81**, 1357-1364(2009).

S2. Want, E. J., Masson, P., Michopoulos, F., Wilson, I. D., Theodoridis, G., Plumb, R. S., Shockcor, J., Loftus, N., Holmes, E., Nicholson, J.K. Global Metabolic Profiling of Animal and Human Tissues via UPLC-MS. N*ature Protocols* **8**, 17-32(2013).
