# Supplementary figures and images for: Antioxidant and anti-inflammatory function of walnut green husk aqueous extract (WNGH-AE) on human hepatocellular carcinoma cells (HepG2) treated with t-BHP
Source: PLoS One. 2025 Jan 27;20(1):e0318005. doi: 10.1371/journal.pone.0318005 (PMC11771903; doi:10.1371/journal.pone.0318005)

**Graphical Abstract**


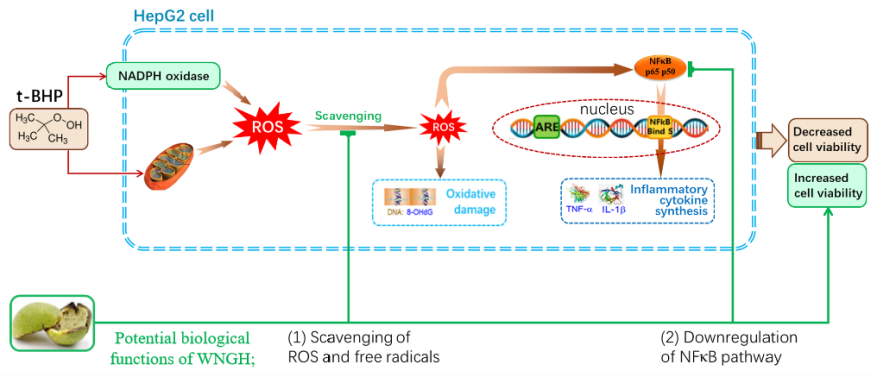

Supplement: S1 Graphical abstract — (DOCX) [file pone.0318005.s005.docx]
